# Communicating the results of risk-based breast cancer screening through visualizations of risk: a participatory design approach

**DOI:** 10.1186/s12911-024-02483-6

**Published:** 2024-03-18

**Authors:** Inge S. van Strien-Knippenberg, Hannah Arjangi-Babetti, Danielle R. M. Timmermans, Laura Schrauwen, Mirjam P. Fransen, Marijke Melles, Olga C. Damman

**Affiliations:** 1 Department of Public and Occupational Health, Amsterdam Public Health Research Institute, Amsterdam UMC, Vrije Universiteit Amsterdam, De Boelelaan 1117, Amsterdam, The Netherlands; 2 Department of Public and Occupational Health, Amsterdam Public Health Research Institute, Amsterdam UMC, University of Amsterdam, De Boelelaan 1117, Amsterdam, The Netherlands; 3 Industrial Design Engineering, Delft University of Technology, Delft, The Netherlands

**Keywords:** Risk communication, Risk visualizations, Risk-based screening, Cancer screening, Breast cancer, Educational material, Informed decision making, Participatory design

## Abstract

**Background:**

Risk-based breast cancer (BC) screening raises new questions regarding information provision and risk communication. This study aimed to: 1) investigate women’s beliefs and knowledge (i.e., mental models) regarding BC risk and (risk-based) BC screening in view of implications for information development; 2) develop novel informational materials to communicate the screening result in risk-based BC screening, including risk visualizations of both quantitative and qualitative information, from a Human-Centered Design perspective.

**Methods:**

Phase 1: Interviews were conducted (*n* = 15, 40–50 years, 5 lower health literate) on women’s beliefs about BC risk and (risk-based) BC screening. Phase 2: In three participatory design sessions, women (*n* = 4–6 across sessions, 40–50 years, 2–3 lower health literate) made assignments and created and evaluated visualizations of risk information central to the screening result. Prototypes were evaluated in two additional sessions (*n* = 2, 54–62 years, 0–1 lower health literate). Phase 3: Experts (*n* = 5) and women (*n* = 9, 40–74 years) evaluated the resulting materials. Two other experts were consulted throughout the development process to ensure that the content of the information materials was accurate. Interviews were transcribed literally and analysed using qualitative thematic analysis, focusing on implications for information development. Notes, assignments and materials from the participatory design sessions were summarized and main themes were identified.

**Results:**

Women in both interviews and design sessions were positive about risk-based BC screening, especially because personal risk factors would be taken into account. However, they emphasized that the rationale of risk-based screening and classification into a risk category should be clearly stated and visualized, especially for higher- and lower-risk categories (which may cause anxiety or feelings of unfairness due to a lower screening frequency). Women wanted to know their personal risk, preferably visualized in an icon array, and wanted advice on risk reduction and breast self-examination. However, most risk factors were considered modifiable by women, and the risk factor breast density was not known, implying that information should emphasize that BC risk depends on multiple factors, including breast density.

**Conclusions:**

The information materials, including risk visualizations of both quantitative and qualitative information, developed from a Human-Centered Design perspective and a mental model approach, were positively evaluated by the target group.

**Supplementary Information:**

The online version contains supplementary material available at 10.1186/s12911-024-02483-6.

## Introduction

Population-based Breast Cancer (BC) screening is prevalent in almost all European countries [1]. The aim of population screening is early BC detection, which offers less burdensome treatment [2]. However, there is no consensus on the benefit of screening on BC mortality reduction and overall mortality reduction (e.g., [3]. Population-based BC can also cause harms, such as false positives, overdiagnosis and overtreatment [4]. In the Netherlands, population-based BC screening is offered biennially to women between 50–75 years. In the near future, this screening may become more stratified or ‘risk-based’, i.e. based on a classification of women in risk categories built on a risk prediction model [5, 6]. The rationale is that risk-based screening likely improves the balance between screening benefits and harms for each risk category [7, 8] but research into risk-based screening is still ongoing. For example, risk-based BC screening could mean that women at relatively high risk are screened more often and those at relatively low risk less often, compared to women at medium risk. Also, women with dense breast tissue could be offered an MRI instead of a mammogram, because mammograms are less effective in detecting abnormalities in dense breast tissue [9]. Although it is not yet clear whether risk-based screening will be implemented and what it will look like, for the current study we assumed that women will receive a mammogram and also complete a questionnaire that assesses some of the key risk factors (e.g., age of first menstruation and family history).

Women are generally positive about risk-based cancer screening and about receiving stratified risk information [6, 10–12], however, it poses new questions for information provision and risk communication. Balanced risk communication in cancer screening is already complex, as screening statistics are difficult to interpret by the lay public, the idea of potential harms in the context of preventive health actions can be counterintuitive [13], and messages about false-positive test results have proven difficult to assess [14, 15]. In addition, people are generally positive about health screening [16], women often overestimate the benefits of BC screening [17], are unaware of potential harms, such as overdetection [18, 19], and often overestimate their risk of developing BC [20]. For people with lower Health Literacy (HL) or numeracy, it is even more difficult to understand this risk information [21–24]. Adequately understanding the results of risk-based screening may be even more difficult, as it may involve, for example, classification into a risk category, understanding the implications of the corresponding screening interval and method, and a numerical message about how many women will develop BC in each risk category. One study using hypothetical personalized survival statistics for BC and prostate cancer showed that people needed supporting information to correctly interpret the personalized statistics provided [25]. Another complicating factor in risk-based BC screening in the Netherlands is that, in contrast to some other screening contexts with personalized risk information, e.g., prenatal or genetic screening, it does not include personal contact with a healthcare provider who can explain the risk information.

To support people’s comprehension of numerical risk information, visualizations can be used [26–29]. Visualizations of risk information can facilitate interpretation by, for example, attracting attention, making part-to-whole relations visible, and giving affective meaning to abstract numbers [27, 29]. For those with lower HL or numeracy, this can be particularly helpful, because reducing cognitive effort and making information more meaningful is especially important to them [30, 31]. However, visualizations do not always increase comprehension beyond numerical formats [32, 33], and visualizations can also be misinterpreted, or cause information overload, distraction, unwanted emotional responses, or even communicate wrong ideas [34–36]. To be effective, visualizations should simplify complex information, be intuitive, take into account people’s existing beliefs and experiences (i.e., the information presented should build on knowledge and experiences people already have so that they can relate new information to what they already know) [37], and align with people’s mental schema for graphs [38]. In addition, personalized risk information may not always easily be accepted by laypersons because the personalized risk often does not match their own perception of their individual risk [39]. This may be due to, for example, a reliance on existing beliefs about the importance of particular risk factors in the prediction model [40]. These existing beliefs, or so-called ‘mental models’, of laypersons should be taken into account when developing information materials, since they shape how new information is processed [41–43]. Information not congruent with people’s existing knowledge could be evaluated as irrelevant [44] or may hamper correct understanding [13]. Understanding the 'mental models' helps to design information that takes into account what people find important while at the same time filling knowledge gaps and correcting misconceptions.

Visualizations cannot only be used for numerical information but also for more ‘qualitative’ information, such as an infographic in which patterns, processes, and relations between concepts can be presented [36]. This may offer opportunities to communicate the information involved in risk-based screening, for example, the interplay of different risk factors in developing BC, or the relation between a particular risk category and the advised screening interval/method.

From a Human-Centered Design approach, it is argued that participatory design, actively involving end users from the start and throughout the design process, helps to develop information materials that actually match the needs of end users [45, 46]. While it is common practice to evaluate health information materials through user testing, the content is usually based on expert input only, rather than also building on the perspectives and existing beliefs of the target group. Previous studies yielded valuable knowledge about women’s perception of BC risk (e.g., [47, 48]) and risk-based BC screening (e.g., [11, 12]). For example, most women overestimate BC risk and have positive attitudes towards risk-based BC screening, where they would accept an increase in screening frequency, but a decrease in screening frequency in case of a low risk is controversial. However, to our knowledge, previous studies generally did not report on translation into actual risk communication materials and in particular supporting visualizations, nor did they focus on women with lower HL [23].

Therefore, this study aimed to develop information materials, including risk visualizations of both quantitative and qualitative information, on risk-based BC screening results for women with varying levels of HL. We did so starting from two approaches: the ‘mental model approach’, focused on capturing existing beliefs and knowledge about an issue involving risk, and ‘Human-Centered Design’, which starts from the actual needs and perspectives of end users and where end users are actively involved in the design process from the start. Specifically, we aimed to: 1) investigate women’s existing beliefs and knowledge regarding BC risk and (risk-based) BC screening from the perspective of implications for information development; and 2) develop novel informational materials for the communication of the screening result in risk-based BC screening, including risk visualizations of both quantitative and qualitative information, through participatory design. The perspectives of both women with lower HL and higher HL were captured across these two aims.

## Methods

### Design

This study used a qualitative approach, starting from the ‘mental models approach’, developed by Morgan, Fischhoff [42], and ‘Human-Centered Design’, specifically participatory design as described by Sanders and Stappers [46]. Therefore, the research team was multidisciplinary with researchers with backgrounds in psychology, health education, and Human-Centered Design. The study consisted of three phases: 1) interviews to investigate prior beliefs and knowledge of women and assess their needs and implications for information development; 2) participatory design sessions to develop novel informational materials; and 3) user-tests to test the informational materials. Parallel to 2) and 3), two experts in risk-based cancer screening and cancer epidemiology, were consulted throughout the development process to ensure evidence-based information content. In 3) five other experts in risk perception and communication and Dutch BC screening assessed the information materials. A plain language specialist translated informational materials to a reading level up to sixth grade. The interviews were conducted between late 2019 and early 2020. The participatory design sessions started in March 2020. Due to Covid-19 restrictions, the participatory design sessions were held online from the second session onwards, as were the user-tests. The Miro program and Zoom were used. Interviews, participatory design sessions, and user-tests were conducted in Dutch. The information materials were also designed in Dutch and translated into English for scientific reporting.

### Participants

Participants in the interviews (*n* = 15) and first three participatory design sessions (*n* = 4, *n* = 6, and *n* = 2) were women without breast abnormalities aged 40–50 years, not yet invited for the current Dutch BC screening program but soon to be eligible. They were recruited by convenience and snowball sampling, starting from the researchers’ own network, and through an online panel (Flycatcher Internet Research; ISO-20252, ISO-27001 certified). The same women participated in the first three sessions, with two additional women participating in the second and third sessions. The fourth and fifth sessions had a more evaluative character in which we wanted to include perspectives of women who had already participated in the current Dutch BC screening; they were between 54–62 years old. In consultation with the Dutch Breast Cancer Association (BVN), we decided to include the perspective of both women without breast abnormalities (session four) and women with breast abnormalities (session five). Participants in the fourth session (*n* = 2) were recruited through convenience/snowball sampling and in the fifth session through the BVN (*n* = 2). Women who participated in user-tests (*n* = 9) were aged 40–74 and recruited through convenience/snowball sampling. Five experts were consulted to assess the information materials.

### Procedure and variables

#### Phase 1: interviews

Interviewees were informed about the study aim; after providing informed consent, the interview started with questions about socio-demographic background. Subsequently, questions were posed about their knowledge, beliefs (‘mental models’) and intention regarding the current Dutch BC screening. Also, questions were posed about individual and general risk perception and BC risk factors. The interviewer then briefly explained the concept of risk-based BC screening and asked about the women’s beliefs and intention regarding risk-based BC screening. Finally, women’s more explicit information needs were assessed. The interview guide was based on prior studies regarding the introduction of risk-based breast cancer screening [11, 49, 50] and is provided in Additional file 1. To assess participants’ HL, interviewees completed the Dutch version of the Newest Vital Sign (NVS-D) [51]. Each interview lasted approximately one hour and women were rewarded with a 20-euro voucher.

#### Phase 2: participatory design sessions

To assess the participatory design participants’ level of HL, they completed the Dutch version of the Functional Communicative and Critical Health Literacy Scales (FCCHL-D) [52, 53]. Prior to the first session, women received a sensitizing booklet (Additional file 2) to activate memories and experiences related to previous health checks [46]. The sensitizing booklets were not handed in but were kept by the women themselves. During the first session, women were asked about beliefs toward the current Dutch BC screening. Using a timeline on the wall displaying the current screening procedure (from invitation to screening result), women were asked to express the perceived goal, their expectations, and feelings at each step. Subsequently, it was explained what potential future risk-based BC screening can entail. The 5W1H method (i.e., who, what, why, where, when, and how questions) was used to gain insight into ideas and expectations that this new screening evoked [54]. After a break, women reflected on the communication of risk-based screening results (e.g., how can the result be presented). For this purpose the H2 method (i.e., how to..) was used, for example, women were asked to indicate how one can convey the results of risk-based BC screening in a clear way or a friendly way and how a low/medium/high risk can be communicated. Based on the ideas generated, women worked in pairs to create and pitch a poster with a concept for communicating the screening result.

In the second session, women reflected on potential benefits and harms of risk-based BC screening. They first did this individually and later in the group. Harms and benefits were explored further to understand underlying beliefs and values. The ladder of abstraction method was used to identify the underlying concepts. This method means that when an answer is given, the ‘why’ question is asked. This results in a higher level of abstraction each time. Participants then evaluated, redesigned, and discussed four draft prototypes of information materials to communicate the screening result, which included several risk visualizations of both quantitative and qualitative information.

The third session was dedicated to how participants interpreted the risk visualizations, which a professional designer had further developed based on the previous sessions, and to use their input in visualizing complex ‘risk concepts’. First, women explained what they thought was the message of six risk visualizations: a) the classification into risk categories; b) the associated absolute risk displayed through an icon array; c) the number of Dutch women in each of the risk categories; d) an overview of the false positives and negatives in risk-based BC screening; e) pictograms related to BC risk factors; and f) benefits and harms of risk-based BC screening (see Additional file 3 for the visualizations). Following a group discussion, participants sketched risk visualizations themselves for three textual risk messages related to: a) BC risk factors; b) the four risk categories with the absolute risk of the medium risk category; and c) the reliability of risk-based screening.

In the fourth and fifth sessions, the risk visualizations resulting from the previous sessions were evaluated by two different groups of women. Furthermore, the underlying goals and values driving women’s needs for certain information were further explored using the ladder of abstraction method. Women who participated in the fourth or fifth session received a sensitizing booklet in advance (Additional file 4). Each session lasted 2–2.5 h and women were rewarded with a 20-euro voucher per session.

#### Phase 3: user-tests

Based on the results of the first two phases, the informational materials communicating the risk-based BC screening result were further developed by a professional designer from the research team. Participants were informed about the study aim, and after providing informed consent they were shown the prototypes. While viewing them, participants were asked to think aloud (i.e., verbalize their thoughts). Also, several experts were consulted to evaluate the informational materials via e-mail.

### Analysis

Interviews were transcribed verbatim and analysed inductively using open and axial coding in the software program MAXQDA. Three transcripts were analysed independently by three researchers and discussed during a meeting (researchers HB, OD, MF). During this meeting, the initial codes and coding tree were identified based on these three interviews. Subsequently, all interviews were coded by using these axial codes (researcher IS) and new emerging codes were discussed in regular research meetings (researchers IS, OD). Based on the results, main themes were identified in a thematic analysis (researchers IS, OD). Subsequently, implications of these themes for information development were determined within the project team (researchers HB, OD, MF). For analysing the participatory design sessions, the three-phase structure for generative data analysis was used [55]. According to the first phase of the three-phase structure for analysis, the researchers documented their findings and impressions immediately after each participatory design session. The second phase consisted of analysing the audio-recording, the created materials (e.g., posters and risk visualizations), and the notes that were taken during the participatory design sessions. In the third phase, the main findings were identified and discussed by the researchers and a professional user experience designer (researchers HB, LS, OD). This process was followed after each participatory design session. Findings were incorporated into information prototypes for the next participatory session/user-tests. Insights and the development process of the informational materials were documented, as recommended for generative research [46]. User-tests were summarized and implications for further development and improvement of informational materials, including risk visualizations, were extracted.

### Ethics

This study was exempted from review by the medical research ethics committee of Amsterdam UMC, location VUmc (FWA00017598) following local regulatory guidelines/standards for human subjects protection in the Netherlands (Medical Research Involving Human Subjects Act). Participants from all three phases provided informed consent.

## Results

### Participant characteristics

Participant characteristics of phase 1 – interviews – and phase 2 – participatory design sessions – are displayed in Table 1. Nine women aged 40–74 with varying educational levels participated in the third phase (user-tests).

**Table 1:** Characteristics of participants from Phase 1 – Interviews and Phase 2 – Participatory design sessions

	**Phase 1 –Interviews**	**Phase 2 – Participatory design sessions**			
	**Interviews (*n* = 15)**	**Session 1 (*n* = 4)**	**Sessions 2 and 3 (*n* = 6)**	**Session 4 (*n* = 2)**	**Session 5 (*n* = 2)**
Demographics					
Age (years), mean (SD)	43.9 (2.6)	43.5 (3.4)	44.2 (3.8)	55.5 (2.1)	58.0 (5.7)
(Youngest-oldest)	40 – 48	40 – 48	40 – 50	54 – 57	54 – 62
Educational level					
Low^a^	1 (6.7%)	0 (0%)	0 (0%)	0 (0%)	0 (0%)
Middle	9 (60.0%)	1 (25.0%)	3 (50.0%)	0 (0%)	0 (0%)
High	5 (33.3%)	3 (75.0%)	3 (50.0%)	2 (100%)	2 (100%)
Health Literacy*					
NVS-D^b^ – low	5 (35.7%)				
NVS-D – high	9 (64.2%)				
FCCHL-D^c^ – low		2 (50.0%)	3 (60.0%)	1 (50.0%)	0 (0%)
FCCHL-D – high		2 (50.0%)	2 (40.0%)	1 (50.0%)	2 (100%)

*SD* Standard deviation

^*^In Phase 1 one HL score is missing; in sessions 2 and 3 of Phase 2 also one HL score is missing

^a^Low education = primary education or pre-vocational secondary education; middle education = secondary vocational education; high education = university of applied sciences or university

^b^Newest Vital Sign in Dutch, which contains six questions regarding the interpretation of information on an ice cream nutrition label, with 4 points or less being defined as having low HL [51]

^c^Functional Communicative and Critical Health Literacy Scales in Dutch, which contains fourteen items measuring subjective skills on a 4-point scale. The total score is the average, ranging from 1 (low HL) to 4 (high HL) [52], with 3 points or less being defined as having low HL [53]

### Phase 1 – Interviews: main themes

The following section describes the four main themes emerging from the interviews, along with their implications for information development. Participants with higher and lower HL were interviewed, but no differences were found in the main themes. Table 2 provides the themes with participants’ quotes. The codes used to analyze the transcripts are included in Additional file 5.

**Table 2:** Overview of themes and sample quotes from Phase 1 – Interviews

Sample quotes	Interviewee
**No reflection on screening benefits and harms; potential harms are seen as disproportionate to the main perceived benefit of early detection of BC, but self-examination is also considered important**	
[About BC screening] If I were to be screened, I think that would bring some reassurance. That I would just know that I did everything I could to keep an eye on myself, but that it is also well monitored from the outside	43 years, high education, unknown HL
[About the main benefit of BC screening] If there is an abnormality, you have an earlier chance of discovering it and you have a better chance of surviving it	43 years, middle education, high HL
[About BC] I always think of that good friend of mine. Well yeah, I know the impact it had on her life. Still has. She will never be the same again. She has … she has also had the whole hassle, so to speak. She first.. they did chemo first. Then she had surgery and then she also had radiation, so her energy level has gone. She is one year younger than me and she actually has no choice other than to use a mobility scooter	40 years, middle education, high HL
[About the considerations regarding BC screening participation] I think it’s indeed… it’s been on my mind for so long at my age that I … Yeah, my mom and mother-in-law, they all do that, so I don’t know any better than that your turn will come eventually	45 years, middle education, low HL
[About the considerations regarding BC screening participation] I’m more like it’s good, you know that. It is, it’s how it’s supposed to be	43 years, high education, high HL
[About the potential harms of BC screening] Well, not for me, but I know that it causes stress for a lot of people… Just the fact that the bus is in the neighborhood stresses quite a few people out. I think that’s the only disadvantage I can think of	47 years, middle education, high HL
[About the potential harms of BC screening] Yes, I hear that it’s painful. And that’s no reason for me not to do it. Because when I look at my friend, she’s in a lot of pain right now, but that’s because of the reconstruction surgeries. Then I think, then it’s better to have discomfort in advance than the pain caused by perhaps intervening too late	43 years, high education, unknown HL
[About self-examination] I think if I feel something in my own body then I’ll go to the doctor to get it checked. So the chances of getting it are nil. Look, if I didn’t do anything about it, I’d say ok. But I feel my own body and I know exactly what’s right and what’s not… Then I go straight to the doctor if it’s not right	47 years, middle education, low HL
**Risk perception: lacking knowledge about breast density and absolute BC risk**	
[About BC risk] My mom doesn't have it. She's 85, so yeah, I don’t think straight away that I'm in a risk factor or anything	47 years, low education, low HL
[About BC risk factors] But it’s also our prosperity. [Interviewer asks what the interviewee means by prosperity] A rich man’s disease, just excessive drinking, excessive smoking, our Western consumer society	48 years, high education, high HL
[About pregnancy] Well, the mammary glands and all. I mean, yeah, that changes. The nipples themselves change a lot after breastfeeding, for example. Yes, I can imagine that might trigger something. And well, also perhaps the hormones that are produced. Although I can also imagine that nature will also protect mothers a little more	40 years, middle education, high HL
[After the interviewer asks whether breast density is a risk factor] No, I don't expect that. Look, being more flexible, you can feel in depth. But one person has strong muscles and the other weak ones, that's how I look at it	45 years, middle education, low HL
[About general BC risk] You hear breast cancer quite a lot of course. One out of three?	45 years, middle education, high HL
[About personal BC risk] I smoke, yes, then I would expect the risk to be higher. But really a lot higher? A little but not very much	45 years, middle education, low HL
[About personal lifetime risk perception] Well, I guess just 50/50, so 50%. You either get it or you don’t. [Question on risk perception over the next 10 years] Yeah, I think it might be just a little bigger. Then it’s 60/40 because you’re getting older	41 years, middle education, high HL
[About the participant’s personal risk perception] Very unpleasant to think about. I really don’t dare say anything. The idea…, jeez. I wouldn’t say anything about that…, I just think you’ll bring it upon yourself when you.., I think so much…, no I really have no idea!	43 years, middle education, high HL
[Interviewer: Is that lifetime chance greater or smaller than in the next 10 years?] Smaller chance, because it’s a longer period	47 years, middle education, low HL
**Beliefs towards risk-based screening are positive, women believe that it will identify personally modifiable risk factors**	
[About risk-based BC screening] I think it's progress, also partly, how do I explain that, self-awareness, it's nice that you get that chance, that's how I see it. But when you get assigned to a group later on in the future, that’s also, self-responsibility, that's what I actually mean, that you also have to do something about it yourself, so to speak	42 years, middle education, high HL
[About risk-based BC screening] I think the benefits are really the awareness that, that it’s… It’s not our fault at all, but that you can contribute a little bit to a healthy lifestyle. And yeah, that people are made aware of it, or that we can be made aware of it	45 years, middle education, high HL
[About risk-based BC screening] I'm positive… I'm positive about it. (…) Yeah, because to know if you're more at risk or something.. Yeah, those are things you'd want to know anyway. But I personally think the risk is small, so…But to get it…[Interviewer: confirmed?] Confirmed I.. maybe I would do it then	47 years, middle education, low HL
[About the lower screening interval for those at low risk] Less often, I would think it’s not completely fair. I don’t drink, I don’t smoke, I practice a super healthy lifestyle, I’m at a healthy weight, there’s nothing in the family, so I’m assigned to the low. And then suddenly I get to come less often. Then I would be tempted to change that slightly, that screening test. If it’s every five years for everyone and then all of a sudden they tell me you don’t have to come so often, so you do it every seven years, I wouldn’t like that	42 years, middle education, high HL
[About a questionnaire to measure risk factors] The only thing… I always think such a questionnaire is of course just what the person wants to fill in. You always paint a rosier or a worse picture of yourself, so it's always difficult to fill it in very clinically honestly. You can always say 'drink very little' but yeah… 'it's not too bad'. It’s very difficult to say exactly: I really fall within this risk behavior, I think	43 years, middle education, high HL
**Explicit information needs include wanting to know why one has been assigned to a certain risk category and wanting advice on risk reduction**	
[About receiving the result of risk-based BC screening] Yes, especially how those pillars are constructed. What makes someone ‘a high risk’, for example? Why average? Or why low? So that it’s well founded. That the factors that are taken into account when coming to a certain decision are clearly explained. So that you don’t get any horror stories, but just get a clear explanation of why a certain route has been chosen	43 years, high education, unknown HL
[About receiving the result of risk-based BC screening] Yes I would really like some advice on how… what can you do to reduce that risk? And that doesn’t mean that you won’t get it, but it falls into the category that you can do something about it yourself	40 years, high education, low HL
[About receiving the result of risk-based BC screening] And what I ‘d like then [when at high risk], I think I would like it if there was a telephone number or a consultation hour, or somewhere to go or something, or to your own GP, or come to you, for example, to give me advice	43 years, high education, high HL
[About receiving the result of risk-based BC screening] What’s also a good thing in those brochures, an overview of breasts with abnormalities that could indicate breast cancer. I think that’s a very clear, yeah, model. I think that also helps for those intervening years. You can also do something yourself, just feel and see if anything changes in your breasts. And then make that call yourself, not relying entirely on breast cancer screening	40 years, high education, low HL

Low education = primary education or pre-vocational secondary education; middle education = secondary vocational education; high education = university of applied sciences or university

#### No reflection on screening benefits and harms; potential harms are seen as disproportionate to the main perceived benefit of early detection of BC, but self-examination is also considered important

In line with what is already known, 13 women had a positive attitude towards BC screening in general and did not have to think ‘hard’ or ‘long’ about participation; they found it reassuring to participate in the future. They viewed BC as a severe disease and believed that screening detects BC at an early stage, which would decrease treatment intensity and increase the chance of treatment success and survival. When asked about potential harms, nine women said that undergoing a mammogram is painful and four women said that participation could cause anxiety. Overall, however, these harms were considered disproportionate to the harms of potential invasive BC treatments when BC is not detected at an early stage.

Two participants indicated that they did not want to participate in BC screening, because they already practiced self-examination and would go to the GP if necessary. Women who intended to participate in BC screening also indicated the importance of self-examination but believed that BC screening detects abnormalities earlier. Three women said they were afraid of self-examination and therefore preferred BC screening.

In terms of information design, these findings imply that it should be communicated clearly that risk-based screening has a better harm-benefit balance compared to current population screening. This idea will likely not be intuitive or immediately clear to women, as they do not perceive an imbalance in the current trade-off between harms and benefits. In addition, self-examination should also be addressed in the information.

#### Risk perception: lacking knowledge about breast density and absolute BC risk

Twelve women spontaneously and correctly mentioned heredity as one of the key risk factors. When asked about other risk factors, lifestyle was also correctly identified. Other risk factors, mentioned spontaneously or after interviewer probes, were having children, breastfeeding, hormones (including the birth control pill), and age (mainly related to menopause). However, the relationship between having children, breastfeeding, and BC risk was not clear, with some women considering these factors to be risk-reducing and others as risk-increasing. Breast density was not mentioned as a risk factor and when the interviewer asked if this could be a risk factor, the women generally had no idea.

When asked about a Dutch woman’s average BC risk over 10 years (among women of the interviewee’s age), the numerical probability was generally overestimated to be around 30% to 50%. However, two women underestimated this risk and thought it to be around 1%. When asked about their own BC risk, participants often responded with verbal terms such as average, above or below average. Three women said they perceived this as a 50/50 chance, others said they had no idea or felt uncomfortable talking concretely about their own risk. There was confusion about the timeframe of BC risk: some women thought that 10-years and lifetime risk were the same and others thought that lifetime risk could be smaller than 10-years risk. No differences were found between women with lower or higher HL.

For information design, these findings imply the importance of clear communication of absolute (medium) risk with the associated time interval and protective/risk factors. In particular, attention should be paid to breast density, as it plays an important role in risk-based BC screening.

#### Beliefs towards risk-based screening are positive, women believe that it will identify personally modifiable risk factors

As expected, based on previous studies, most participants [13] responded positively to the idea of risk-based BC screening. That personal risk factors would be taken into account that were – in their perception – to a great extent modifiable, such as lifestyle, was especially seen as an improvement. Women’s beliefs seemed to be related to a more general positive attitude towards a healthy lifestyle and taking responsibility for one’s health. Women already seemed positive about a healthy lifestyle and thought that the link between your breast cancer risk and a healthy lifestyle would be extra motivating for a healthy lifestyle. In general, women were also positive about knowing the absolute BC risk belonging to their risk category. Even those who said they did not want to participate in BC screening would reconsider this decision if risk-based screening were to be introduced and would, for example, participate once to come to know their risk.

When asked about potential harms of risk-based BC screening, it was mentioned that a higher than medium risk could cause anxiety. In addition, it was stated that low-risk women should not be screened less often than is currently the case, or at least should be allowed to do so, because otherwise, it would be ‘unfair’ that those with a healthy lifestyle are allowed to screen less often. However, the exact current screening interval was not known to everyone, so this opinion seemed to be more concerned with the idea that you are allowed to screen less often than about the actual interval itself. Another potential harm mentioned was the way risk factors such as lifestyle will be measured, as a questionnaire was considered too subjective.

In terms of information design, the findings stress the importance of explaining that a combination of different risk factors contributes to a woman’s BC risk, rather than a few modifiable risk factors. Information should further be designed in such a way that it will not cause too much anxiety in those assigned to a higher risk category and not cause too negative reactions in terms of feeling disadvantaged in those assigned to a lower risk category.

#### Explicit information needs include wanting to know why one has been assigned to a certain risk category and wanting advice on risk reduction

When asked explicitly to specify their information needs, the importance of knowing why you have been assigned to a particular risk category was often mentioned. Women said that for those at high risk, this explanation should be accompanied by advice on how to reduce BC risk and the opportunity to contact a healthcare professional. Information about the rationale behind risk-based BC screening was found important as well, for example, through layered information. Information about breast self-examination was again mentioned.

In terms of information design, in line with the points discussed above, these results emphasize the importance of stating the reason for classification in a certain risk category, providing advice on reducing risk, and mentioning self-examination.

### Phases 2 and 3: participatory design sessions and user-tests – key insights

**Fig. 1:**
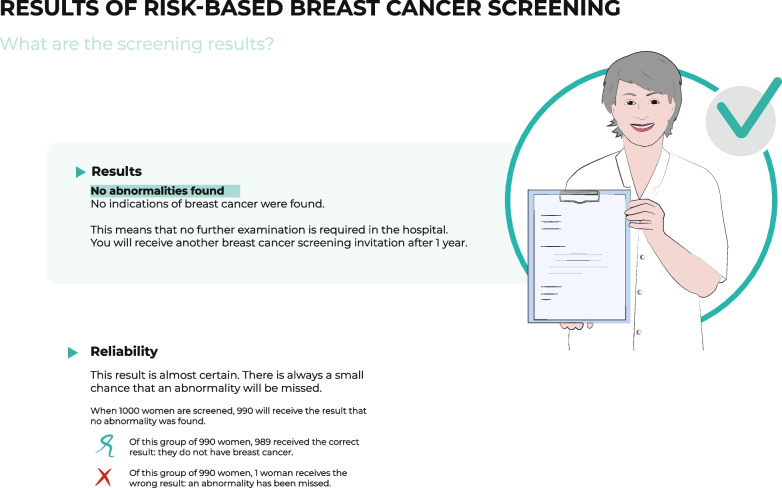
Visualization of the result no abnormality found

**Fig. 2:**
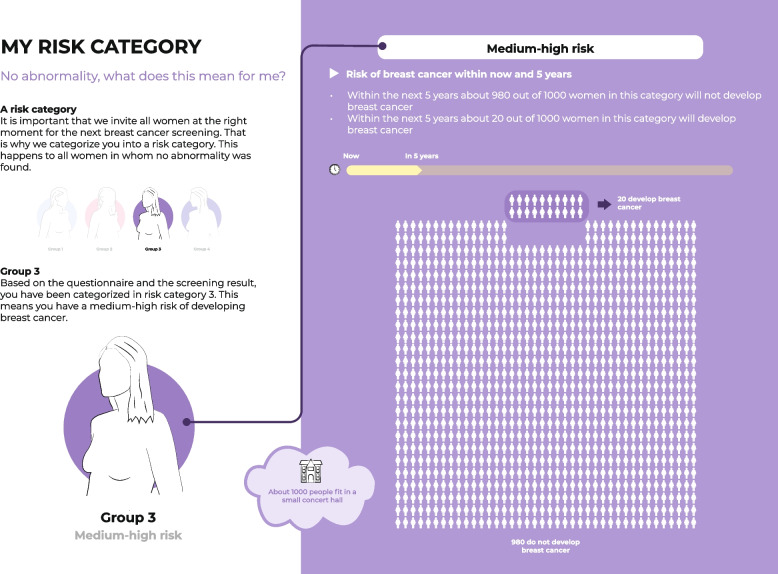
Visualization of the absolute probability information in relation to the risk category

**Fig. 3:**
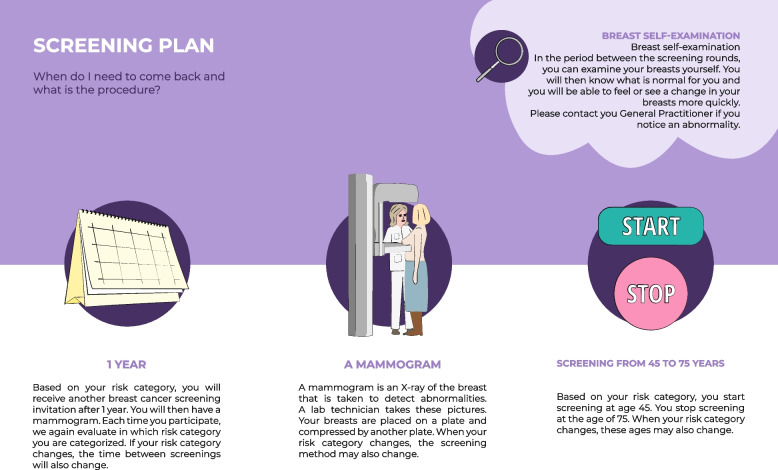
Visualization of the screening plan and information on breast self-examination

**Fig. 4:**
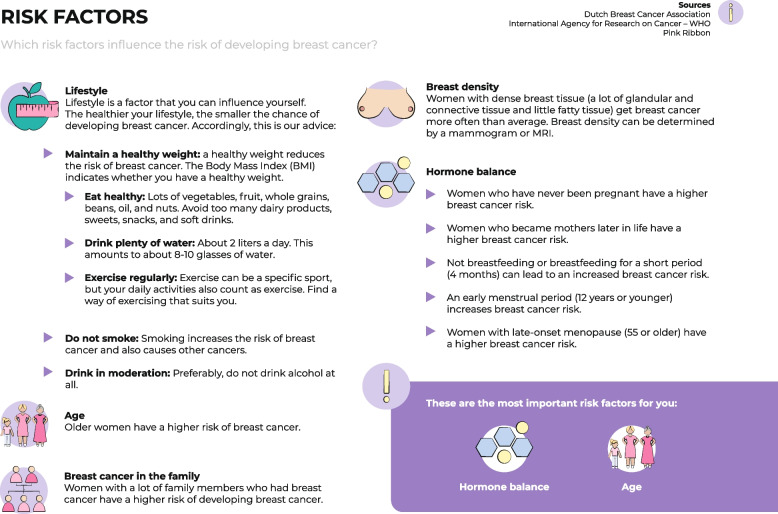
Visualization of the risk factors

**Fig. 5:**
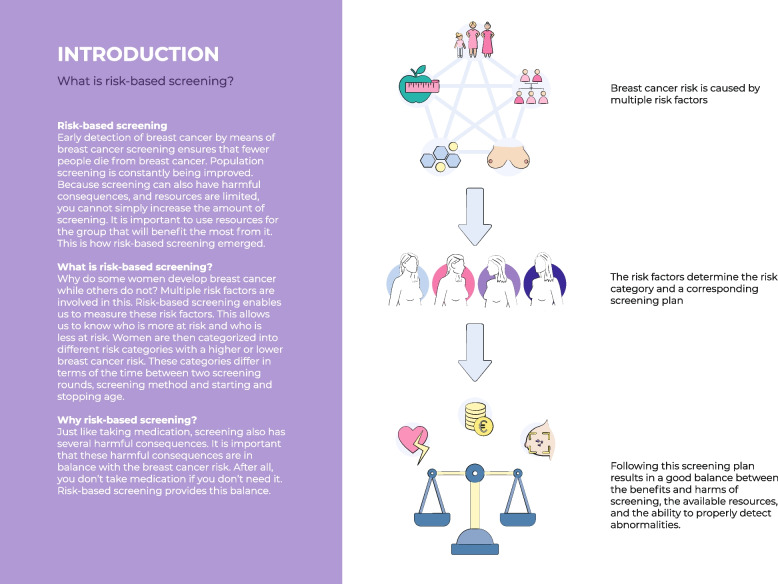
Visualization of the rationale behind risk-based screening

**Fig. 6:**
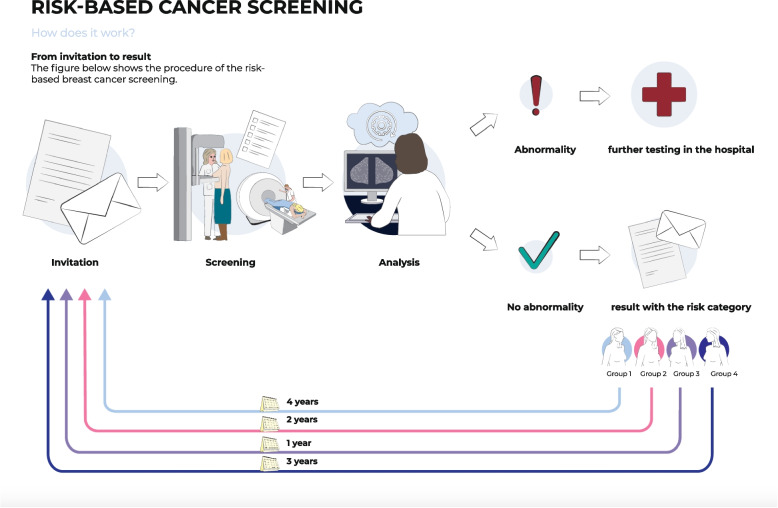
Visualization of the screening procedures of the four risk categories

**Fig. 7:**
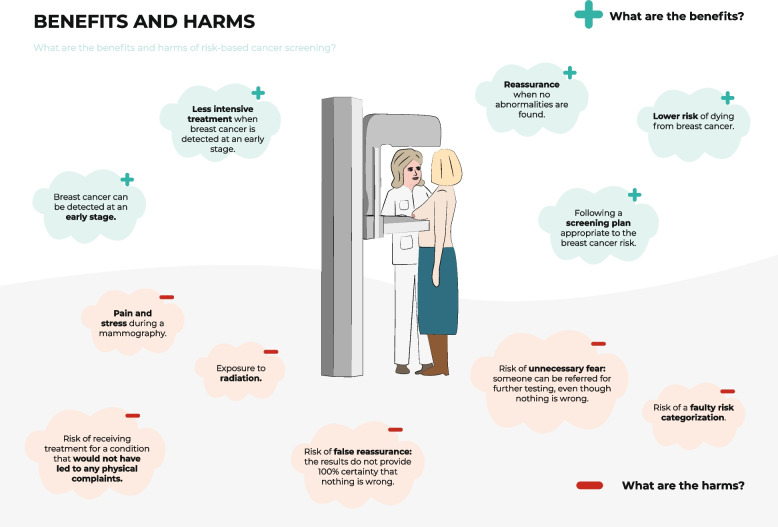
Visualization of the benefits and harms of risk-based screening Note: The materials presented are the materials as they were shown to participants in Phase 3 (user-tests)

The following section describes the main themes emerging from Phase 2. To include a diverse population, participants with higher and lower HL participated in this phase. However, due to the nature of the participatory design sessions, in which many assignments were made in collaboration between women with lower and higher HL, it was not possible to distinguish between the two groups in the analyses. Following each theme, we describe information design implications, in particular for the design of visualizations. We also indicate how the resulting prototypes, as shown for the third risk category in Additional file 6 and Figs. 1, 2, 3, 4, 5, 6 and 7, were evaluated in the user-tests (Phase 3). Table 3 provides the objectives, methods, and specific results per session of Phases 2 and 3.

**Table 3:** Overview of methods and results of Phases 2 and 3—Participatory design sessions and user-tests

Elements, participants, and researchers	Objective	Method	Results and key insights
*Phase 2 – Participatory design sessions*			
Session 1 Women aged 40–48 who had not yet been invited for BC screening (*n* = 4) Researchers (HB, OD, LS)	To gain insight into women’s expectations and feelings regarding the current population-based BC screening	Elicitation of women’s goals, expectations and feelings regarding the different steps in the current BC screening program by using a timeline method	• Invitation does not automatically lead to interest • Difference between inviting to participate and informing about the possibility of participation • Important to know what to expect (screening procedure and result) to reduce tension
	To gain insight into women’s expectations and feelings regarding risk-based BC screening	Elicitation of women’s ideas and expectations that the new risk-based BC screening evokes by using the 5W1H method (i.e., who, what, why, where, when, and how questions)	• More effective and cost-effective with earlier detection in the case of higher than medium risk • Awareness of risk factors, especially lifestyle • Can cause anxiety or false reassurance
	To investigate the information needs regarding risk-based BC screening and how information about risk-based BC screening should be presented	Generating ideas using H2s (i.e. how to …) for the communication of risk-based screening and creating and pitching a poster with a concept for how this information can be presented	• Personal and cordial tone • Advice on how to reduce risk • Terminology should be unambiguous • Explanation of what the risk categories entail • Words and visualizations • Color choice is important and should match the severity • Use of recognizable symbol • Logo to indicate reliability of information source • Preference for receiving a letter, potential additional information online • Possibility to get in touch with someone (e.g., phone, chat) • Personal contact with GP in the case of abnormality • To stimulate participation, choosing your screening location and time is important
Session 2 Women aged 40–50 who had not yet been invited for BC screening (*n* = 6) Researchers (HB, OD, LS)	To gain insight into women’s perceptions of the benefits and potential harms of risk-based BC screening	Individual reflection followed by a group discussion on the benefits and potential harms	Benefits: • More personal • More targeted and cost-effective • Higher participation rate • Researchers can do more research Potential harms: • Anxiety, especially when at high risk • Classification in wrong risk category • Women are pigeonholed • Pressure to live healthier • Too little screening for those at low risk
	To understand the thoughts and values underlying the benefits and potential harms of risk-based BC screening	Using the ladder of abstraction method (i.e., method of moving from concrete to more underlying concepts) to further explore the mentioned benefits and potential harms	• Trust in the classification into risk categories • Anxiety plays a role in information provision; it is important to only receive relevant information
	To examine women’s responses and suggestions for improvement to (draft) risk visualization prototypes	Individual evaluation and redesign of four risk visualization prototypes, followed by a group discussion	• Overview, for example through a flowchart • Important not to have to search for the core of information • Information from general to specific • Layered information • Rationale behind the risk category classification • Advice on modifiable risk factors • Consistency regarding how numbers are displayed • Too much information and too many numbers (e.g., about the other risk categories) creates confusion • Icon arrays are clear • Pictograms must be unambiguous • Visualizations must add value
Session 3 Women aged 40–50 who had not yet been invited for BC screening (*n* = 6) Researchers (HB, OD, LS)	To gain insight into women’s interpretation of different (draft) risk visualizations	Explaining the meaning of six different (draft) risk visualizations	• Different colors to indicate different risk categories is clear • Abstraction in female images is important, otherwise meaning is given to irrelevant details (e.g., breast shape) • Detailed numeric information about BC screening reliability (false positives and negatives) is perceived as too much information • Information about all risk categories causes confusion, anxiety, and preference for an MRI
	To formulate ideas about how women would visualize complex risk information themselves	Designing three risk visualizations based on three textual messages	• Pictograms are helpful to indicate risk factors • It is important to know which risk factors can be influenced • Keep it as simple as possible, preferably only presenting risks from own risk category • Unambiguous information, do not display confidence intervals
Session 4 Women aged 54–57 who had already participated in BC screening, but without any breast abnormalities (*n* = 2) Researchers (HB, OD, LS)	To investigate how women who had already participated in the current BC screening program perceive the (draft) risk visualizations	Explaining the meaning of the (draft) risk visualizations resulting from the first three participatory design sessions and evaluating them	• The colors used to indicate different risk categories must differ sufficiently from each other • Explanation of why someone is classified in a certain risk category is important • What the screening interval entails must be clearly explained • Clear explanation of the influence of risk factors is needed • More abstraction is needed in the female images to avoid giving meaning to irrelevant details • Icon arrays are clear • Information about false positives and false negatives is difficult to understand and seems to be irrelevant
	To understand why women need certain information about risk-based BC screening	Exploring underlying goals and values through the ladder of abstraction method (i.e., method of moving from concrete concepts to more underlying ones)	• Being well-informed about screening reduces uncertainty and increases motivation to participate • Stress and fear can be reasons not to participate
Session 5 Women aged 54–62 who had already participated in BC screening and were diagnosed with breast abnormalities (*n* = 2) Researchers (HB, OD, LS)	To investigate how women who had already participated in the current BC screening program and who were diagnosed with breast abnormalities perceive the (draft) risk visualizations	Explaining the meaning of the (draft) risk visualizations resulting from the first three participatory design sessions and evaluating them	• Information about risk factors can lead to blaming those who are at high risk/have abnormalities • Importance of explaining why someone is classified in a certain risk category and whether this risk category always remains the same • Icon arrays are clear • Prevent information overload, for example, how many women are in a certain risk category in the Netherlands is not relevant for most women • Information about false positives and false negatives can be difficult for those with lower HL • Information about breast self-examination is important • Layered information is important
	To understand why women need certain information about risk-based BC screening	Exploring underlying goals and values through the ladder of abstraction method (i.e., method of moving from concrete concepts to more underlying ones)	• Understanding the harms and benefits of screening enables you to make a well-informed choice on your own • Unfamiliarity can lead to fear • Self-efficacy and having control are important in relation to your health
*Phase 3 – User-tests*			
User-tests Women aged 40–74 (*n* = 9) Researchers (LS, OD)	To examine how the informational materials, including the risk visualizations, resulting from the participatory design sessions are perceived by women	Verbalization of women’s thoughts as they viewed a prototype of the risk-based BC screening informational material	• Risk visualizations can help to see the most important information at a glance • Some pictograms were not clear (e.g., endocrine system and breast density) • Flowchart of the different screening intervals of the different risk categories creates an overview but should be made clearer • Absolute risks of the risk categories are experienced differently • Using metaphors is confusing • Information about breast self-examination is important • Emphasize that risk-based BC screening is based not only on a mammogram, but also on a questionnaire • It should be clear that the high-risk category has an increased risk and that it is not certain that they will get BC • Breast density is an unfamiliar risk factor and must be clearly explained • Indicating reliability of BC screening is fair

#### Positive beliefs towards risk-based BC screening, mainly based on the belief that it will identify personally modifiable risk factors

Women’s beliefs regarding risk-based BC screening resembled those in the interviews. For example, women again emphasized personally modifiable risk factors and did not identify breast density as a key risk factor. However, beyond the positive aspect of awareness of a healthy lifestyle, some feared that emphasizing these risk factors could lead to feelings of shame or even blame from other people. Potential harms of risk-based BC screening that were mentioned were, as in the interviews, anxiety if categorized into high-risk categories, and the prolonged screening interval for those at low risk. Women also emphasized the risks of false reassurance when at low risk and being categorized into the wrong risk category.

Information design took these topics into account by stating the general harms and benefits of risk-based BC screening (Fig. 7) and highlighting that BC risk depends on a complex interplay of various risk factors (Figs. 4 and 5), whereby breast density was emphasized and explained (Fig. 4). However, user testing showed that the pictogram used to represent breast density was not clear and that the concept of breast density needed further explanation. To reduce fear when classified to a high-risk category, the information emphasized that women received a positive screening result through a green check mark (Fig. 1) and by displaying the probability information – still showing a relatively low absolute risk – related to the assigned risk category in an icon array (Fig. 2). In the user-tests, women said that the green check mark was clear and helped them to understand that although there is an increased BC risk, no abnormality was found. However, women still wanted a more explicit description that being at high risk does not mean they will get BC. The designed icon array was perceived to be clear, but participants indicated they had expected higher absolute probabilities.

#### Importance of clear explanation of the rationale and implications of the risk category to be well-informed and feel in control

Women attached importance to receiving an explanation of why they would be classified into a certain risk category, what this risk category exactly entails, and whether this risk category would always remain the same. Women said that this information should include an explanation of the influence of risk factors and the screening interval to increase their confidence in risk-based BC screening.

Self-efficacy and being in control were stressed as important aspects related to health. This was reflected by discussions about the following needs: 1) receiving clear information about benefits and harms of BC screening to make an informed decision; 2) having some control over locations and date of the screening; 3) receiving screening advice on modifiable risk factors to be able to reduce BC risk; 4) being informed about what women can do themselves to detect BC between screenings (i.e., breast self-examination).

In the information design, the rationale behind risk-based screening and the implications of being assigned to a particular risk category were explicitly stated and visualized (Figs. 3 and 5). However, user-tests showed that the visualization of the rationale needed to be clearer (see Fig. 5, the pictograms associated with the scale). Regarding self-efficacy and being in control, the information included advice on a healthy lifestyle and information on breast self-examination (Figs. 3 and 4). The information on risk factors (Fig. 4) appeared to be especially appreciated in the user-tests.

#### Layered, unambiguous, personal information with consistency in numerical format

With regard to risk-based BC screening, women stated that the information they receive would apply to them personally, which could reduce fear and uncertainty, since irrelevant information is left out. In line with this, layered information was recommended by women to avoid overload, but also to support those who want more information. For example, when we provided women with draft (numerical) information on all risk categories, various women said they felt confused and anxious, but at the same time they said they wanted access to this information. Women further emphasized that the total amount of numerical information should be limited (e.g., no confidence intervals, nor numerical information about false positives/false negatives) and that consistent formats should be used (e.g., consistent X in 100 format).

Women preferred receiving the risk-based screening result in a physical letter with the option to get in touch with someone via phone or chat. Women said that the concept of risk-based BC screening created the expectation that it is more personal, and they also expected the information materials to have a warm and personal tone. The language should be simple and the terminology unambiguous; for example, words like positive and negative screening results were considered to be difficult to interpret. Women also mentioned the importance of unambiguity concerning the visualizations. For example, the color choice of the risk categories should match their severity. Green and red should be avoided as they were associated respectively with no risk and definitely getting BC. Women also emphasized that visualizations should add something to textual information, for example by providing an overview through a flowchart.

The information designed used plain language with clear terminology, with a plain language specialist translating all text to a reading level up to sixth grade. Only information related to the assigned risk category was presented. An exception to this was the overview of the screening procedure for all risk categories (Fig. 6) to also meet the needs of those wanting an overview of the total risk-based screening procedures. This was appreciated in the user-tests, but could be improved, according to the women. Also, the visualization of harms and benefits could be improved, for example by grouping all aspects to create more of an overview (Fig. 7). Regarding the risk categories, these were represented by four abstract female figures to avoid interpretation of irrelevant details such as breast shape. Matching colors were used, for example, purple and blue, to avoid signal colors (Figs. 2, 5 and 6).

### Experts’ views: general information on risk factors, precise information on risks

The experts who ensured the accuracy of the information materials emphasized that from an epidemiological perspective, it would only be possible to indicate the extent to which risk factors contribute to BC risk at the population level, rather than on an individual level. It was therefore considered a ‘misconception’ of women that risk-based BC screening would identify individually modifiable risk factors. Moreover, most risk factors included in risk-based BC screening cannot be influenced by the individual and those that can be influenced are lifestyle factors, which are not specifically related to BC but have an overall impact on a person’s health and disease risk. According to the experts, the main advantage of risk-based BC screening is an improvement in the harm-benefit trade-off for each risk category (i.e., at the population level). Experts also suggested several nuances to information content. For instance, to accurately indicate absolute probabilities belonging to each risk category, and to avoid false certainty when presenting whole numbers, they suggested communicating the risks per 1000 women instead of per 100 women. In doing so, they preferred communicating a range rather than a single number (e.g., 16–18 out of 1000 instead of 17 out of 1000). The experts who assessed the resulting information materials in the user-tests had some comments about inconsistency in word choice, the order of risk factors (they suggested mentioning the most important risk factor first), and recommended not using too many numbers.

## Discussion and conclusion

This study aimed to develop information materials, including risk visualizations of both quantitative and qualitative information, on the possible future risk-based BC screening results for women with varying levels of HL. We did so through the mental model and Human-Centered Design approach. Emerging themes across interviews and participatory sessions were the importance of explaining the rationale and implications of risk category assignment, including informing and reassuring women when assigned to a high-risk or low-risk category. It should also be explained that a combination of different risk factors contributes to a woman’s BC risk, rather than a few modifiable risk factors. Resulting information materials, including visualizations of qualitative and quantitative risk information, were positively evaluated.

As in previous research, women in our study were positive towards risk-based BC screening [56]. Women considered a benefit of risk-based BC screening that it would identify personally modifiable risk factors, like lifestyle, and as such would support them in reducing risk. However, experts stressed that, from an epidemiological point of view, the benefit of risk-based screening is a better trade-off between harms and benefits of the different risk categories, i.e., on population level. However, women were not aware of BC screening harms such as false positives or overtreatment, or did not perceive them as real harms, which is in line with previous research (e.g., [18, 19, 57]). Previous research also showed that reducing false positives and overdetection was considered less important to the lay public than saving lives [58]. Compared to the view of experts, women are less aware of the importance of a better harm/benefit balance. It thus seems important to communicate the main idea of risk-based BC screening more clearly as this is not intuitively clear to them.

The importance attached to modifiable risk factors, especially lifestyle, may be related to lifestyle being a controllable factor, unlike, for example, age of first menstruation. The importance women attach to information on breast self-examination may also be related to control. In theoretical models of how laypersons perceive health threats, such as the Common-Sense Model, control is one of the attributes involved in laypersons mental representations [59]. Previous research on BC screening also showed that women value controllable factors because they feel responsible for their BC risk [47]. Controllable risk factors also play an important role in the lay public’s perceptions of the outcomes of risk prediction models [60, 61]. Although experts noted that lifestyle is not a major BC risk factor, but rather a general risk factor, information on risk-based BC screening should address controllable risk factors such as lifestyle, as they are important for women and it is known that people reject information when it conflicts with their prior beliefs/expectations [44, 60, 62]. However, information should also ideally fill key knowledge gaps, e.g. that BC risk depends on a complex interplay of risk factors, which are not all modifiable, and that breast density is one key risk factor involved.

Our study confirmed previous studies that found an overestimation of absolute BC risk [20, 48, 63]. The positive screening attitude, lack of awareness of potential harms, and the overestimation of BC risk, may contribute to the acceptance of a higher screening frequency for women at high risk and a reluctance regarding a potential lower screening frequency for those at low risk, which is also in line with previous research on BC screening [49, 50, 64]. However, our results show that not all women were aware of the current screening interval. These findings indicate that information materials need to include a plain rationale for the screening frequency associated with each risk category, rather than a comparison with the current population-based BC screening. Moreover, it seems vital to clearly communicate the absolute risks associated with the different risk categories, especially so as not to cause too much anxiety among those at higher risk. In the case of the higher risk categories, it is important to emphasize that no abnormalities were found during the screening, since a higher risk does not mean that you will get BC.

Concerning the quantitative risk information associated with the risk category being classified, the use of icon arrays was preferred, which is in line with the International Patient Decision Aids Standards [29]. Although women in the user-tests seemed to understand the risk associated with the risk category, we do not know if women believed this risk information, as understanding personalized health risk information does not automatically lead to a belief that this risk information actually applies to themselves [39, 65]. Existing beliefs (e.g., [40]) and convictions about personally relevant risk factors are important for the acceptance of risk information [60]. However, for information design it is also important to include all relevant risk factors. Therefore, women should be provided with risk information that matches their prior expectations [39, 43, 44], without providing inaccurate risk information. This could include, for example, an explanation of why a certain risk factor that is important according to women -but not from an epidemiological perspective- is not taken into account in the risk-based screening. Paying some attention to this risk factor in the information can ensure a better connection with women’s prior beliefs, while at the same time, misconceptions about the importance of this risk factor can be corrected.

Regarding the qualitative information, this study has shown that it is possible to visualize complex risk information about risk-based screening, such as the different risk factors involved. When designing visualizations, details and ambiguity should be avoided in order not to evoke mistaken ideas. For instance, women gave meaning to the size/shape of the breasts and posture of the female image while the aim of the visualization was purely intended to indicate risk categories. Developed information materials, including risk visualizations, were generally well-evaluated by the target group and experts. However, preference for information formats does not always correspond with better understanding [30]. In the user-tests, comprehension was examined but this, as stipulated, does not automatically lead to believing the risk-based BC screening result [44, 60, 62]. Further research should therefore focus on understanding and believing the information (i.e., believing that the risk applies to you personally and that risk-based BC screening is an improvement). Further research is also needed to investigate whether people with lower HL indeed benefit from the designed risk visualizations. Although recently more research has been conducted on informing women about their risk of breast cancer when participating in breast cancer screening [66, 67], it is important that research is conducted on information materials when inviting women to participate in risk-based BC screening. For example, the information in the invitation for risk-based BC screening after classification in a specific risk category will need to contain more specific information about the exact harms and benefits associated with the relevant risk category than is the case in the results folder. Research into how this information can be communicated understandably is recommended, as this information can be complex for the general population.

### Strengths and limitations

A study strength is the use of ‘mental model’ interviews to gain insight into women’s knowledge and beliefs with participatory design sessions from a Human-Centered Design perspective. This enabled us to develop information materials and risk visualizations in a participatory way. We managed to include women with higher and lower HL (35.7% low HL in the interviews and 46% on average in the participatory design sessions). However, according to the researchers’ observations, participants belonging to ethnic minorities in the Netherlands were not well represented during the three phases. It should be noted that HL was assessed differently in the interviews (NVS-D) compared to the participatory design sessions (FCCHL-D). During the interviews the NVS-D was administered individually and during the participatory design sessions HL was assessed using a self-administered questionnaire. According to the researchers, the NVS-D was less suitable for administering in this way because it could be experienced as taking an exam. Another limitation of this study is that it focuses on the design and evaluation of the information materials of the results of risk-based BC screening and that the understanding of the materials was only tested with think-aloud interviews in the user-tests.

### Conclusion

Information materials about risk-based BC screening should explain the rationale and implications of risk category assignment, including informing and reassuring women when assigned to a high-risk or low-risk category. In doing so, it is important to explain that a combination of different risk factors contributes to a woman’s BC risk, rather than a few modifiable risk factors. The ‘mental model approach’ and Human-Centered Design offered opportunities to design information materials including risk visualizations from the perspective of the target group and their prior beliefs, increasing the likelihood that the information will be processed and understood.

### Supplementary Information

**Supplementary Material 1.**

**Supplementary Material 2.**

**Supplementary Material 3.**

**Supplementary Material 4.**

**Supplementary Material 5.**

**Supplementary Material 6.**

## Data Availability

Raw data of the interviews are available on request from the corresponding author (in Dutch). Raw data of the participatory design sessions have not been shared, given the privacy of participants and ethical restrictions.

## Abbreviations

BC: Breast Cancer
HL: Health Literacy
