# Supplementary material for: Communicating the results of risk-based breast cancer screening through visualizations of risk: a participatory design approach
Source: BMC Med Inform Decis Mak. 2024 Mar 18;24:78. doi: 10.1186/s12911-024-02483-6 (PMC10949766; doi:10.1186/s12911-024-02483-6)
Supplement: Supplementary file 1 — Supplementary Material 1. [file 12911_2024_2483_MOESM1_ESM.pdf]

# Interview guide

- *Thank the interviewee for wanting to participate.*
- *Repeat the information about the study aim as stated in the Informed Consent form.*
- *Check if there are any questions.*
- *The participant gives informed consent.*
- *Mention again that the interview is being audio-recorded and turn on the audio-recorder.*

- *Always ask for the reasons/why behind the answers.*
- *Probes: Can you tell more? Is there anything more that comes to mind? Can you elaborate/explain that a bit more?*

**Socio-demographic background**

1. Can you tell us something about yourself?
    - What is your family situation?
    - How old are you?
    - What is you highest level of completed education?
    - What do you do in daily life (work, education)?

**Knowledge/beliefs/intention to participate current population-based breast cancer screening**

2. What do you know about breast cancer screening?
    - Do you know from what age on you will be invited?
    - Can you tell me what you know about what happens during the screening?
    - How did you get this knowledge about the breast cancer screening?
3. Why do think there is a breast cancer screening program in the Netherlands?
    - What do you think is the goal of breast cancer screening?
    - What are the potential advantages?
    - What are the potential disadvantages?
4. If you would be invited for the current screening program, would you participate?
    - Why? / Why not?
    - What considerations do you make? Which aspects play a role in your decision?
    - Which feelings / emotions does (participating in) breast cancer screening evoke?

**Individual and general risk perception and BC risk factors**

What comes to mind when you think about breast cancer?

5. How many women in the Netherlands do you think will get breast cancer?
    - How many women in the Netherlands will get breast cancer during their lifetime? (X out of 10/ X out of 100)
    - What do you think of this risk? (small, moderate, large)
    - How many women in the Netherlands will get breast cancer within the next 10 years?
    - What do you think of this risk? (small, moderate, large)

6. What comes to mind when you think about your own breast cancer risk?
    - Can you also express this risk in X in 100 women?
    - Is your risk the same as, or smaller/larger compared to Dutch women your age? Why?
7. Can you tell us why some women are more likely to get breast cancer than others?
    - What might cause breast cancer do you think?
    - Do you know any more risk factors?
    - To what extent do you think <risk factor> can influence the development of breast cancer? Why? / Why not?
      *If not mentioned, mention the risk factors: age/aging; previous breast disease; genetic predisposition; dense breast tissue; lifestyle; hormones/hormonal influences; having children; environmental influences.*

**Explanation of risk-based breast cancer screening**

*The emergence of prediction models will make it possible to make the breast cancer screening more 'personalized' in the future. This means that women will be categorized into risk categories. In population-based breast cancer screening, everyone receives the result whether or not an abnormality has been found. The risk-based screening leads to the result 1) no breast abnormality has been found; 2) a personalized risk profile (low, average or increased risk); 3) a subsequent recommendation for a screening interval in the future.*

*Show the image below.*

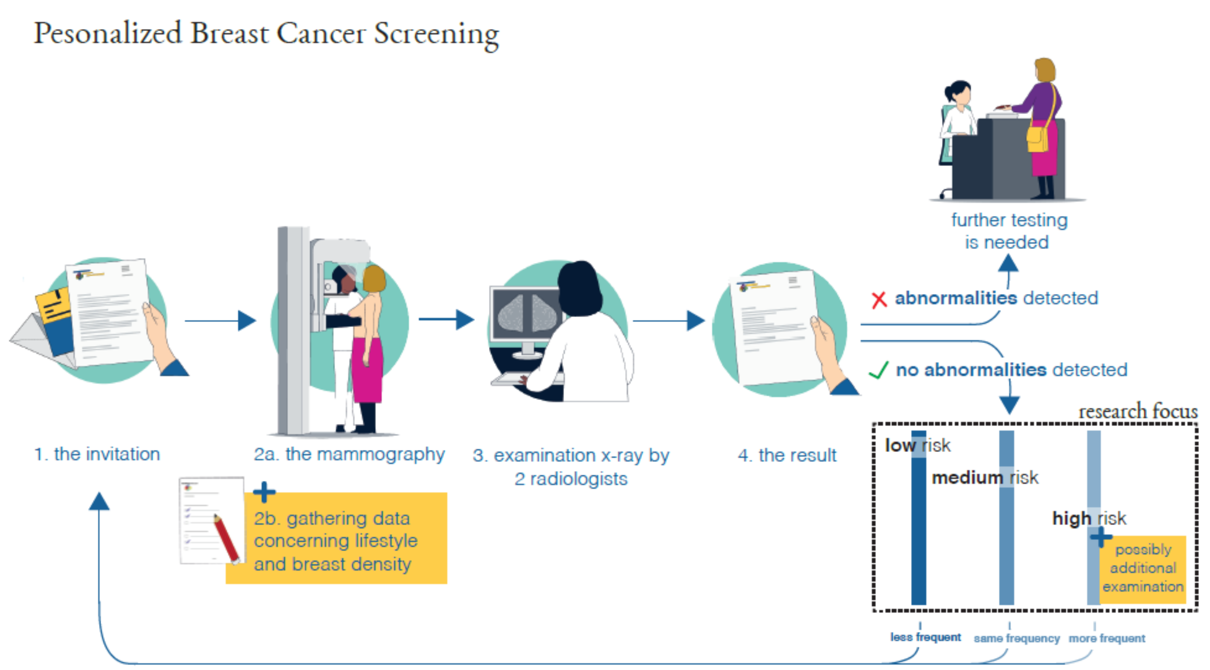

**Beliefs and intention regarding risk-based breast cancer screening**

8. What comes to mind when you hear about this risk-based breast cancer screening?
    - According to you, what might be the advantages of risk-based breast cancer screening?
    - According to you, what might be the disadvantages of risk-based breast cancer screening?
    - Do you think risk-based breast cancer screening would be an improvement over population-based breast cancer screening? Why? / Why not?
9. If you would be invited for such risk-based BC screening, would you participate?
    - Why? / Why not?
    - What considerations do you make? Which aspects play a role in your decision?
    - What would participation in risk-based screening mean for you?
    - What would categorization into a low/high risk category mean for you?

**Explicit information needs**

10. If risk-based BC screening would be introduced in the future, what information would you like to receive about this screening program?
    - What would you like to know about your result?
    - Do you think you would want to know your risk category?
    - How would you like to receive this information?

- *Check if there are any remaining questions.*
- *Complete the Health Literacy questionnaire.*
- *Thank the interviewee.*
- *Hand over the voucher.*
