# Supplementary material for: Communicating the results of risk-based breast cancer screening through visualizations of risk: a participatory design approach
Source: BMC Med Inform Decis Mak. 2024 Mar 18;24:78. doi: 10.1186/s12911-024-02483-6 (PMC10949766; doi:10.1186/s12911-024-02483-6)
Supplement: Supplementary file 2 — Supplementary Material 2. [file 12911_2024_2483_MOESM2_ESM.pdf]

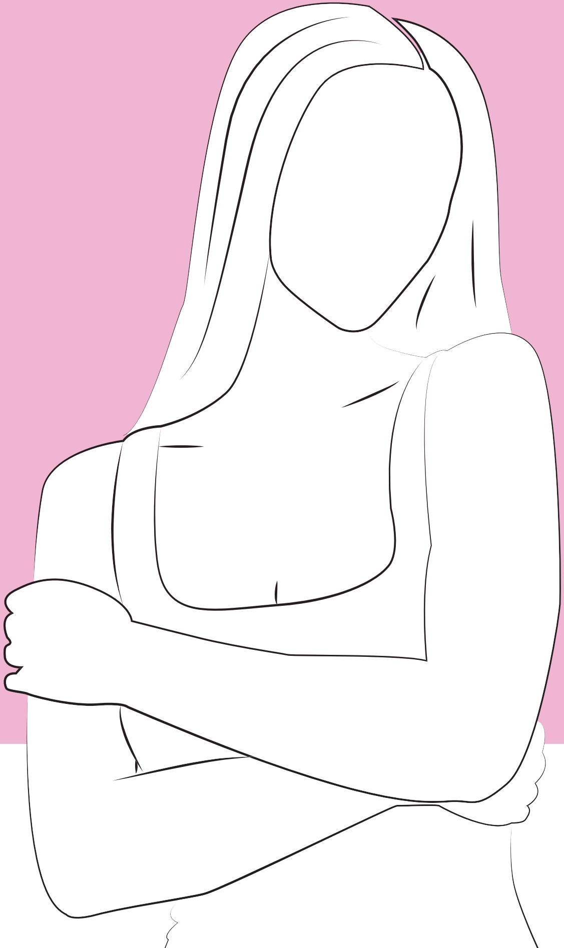

Preventive examination and screening

My experiences and expectations

Amsterdam UMC

INTRODUCTION

Thank you for participating in the creative session on breast cancer screening. This booklet consists of 2 short assignments for you to complete at home in preparation for the session. Do not forget to bring this booklet to the session.
There are no right or wrong answers. We are curious about your own experiences and opinion!
If you have any questions, you can always contact us:
:

06-12345678
example@amsterdamumc.nl

06-12345678
example@amsterdamumc.nl

ABOUT ME

Name: ______________________________

Age: ______________________________

Place of residence ______________________________

Education/profession/background: ______________________________

____

ASSIGNMENT 1: MY EXPERIENCE WITH PREVENTIVE EXAMINATION AND SCREENING

Preventive examination or screening is a medical examination of people who have no health issues. The research is aimed at detecting a disease, a hereditary predisposition for a disease, or risk factors that increase the risk of a disease.

Assignment 1A: I have experience with the following types of preventive examination/ screening:

- ☐ I have no experience with this (go to section 1B)
- ☐ Population screening for cervical cancer (go to part 1C)
- ☐ Screening during pregnancy (blood test, Down's, Edwards' and Patau's screening, or 20-week ultrasound) (go to section 1C)
- ☐ Preventive examination of cardiovascular diseases (go to section 1C)
- ☐ Other preventive examination/screening, namely: (go to part 1C) ....

Assignment 1B: I have no experience with preventive examination/screening, because...:

- ☐ I have never received an invitation for this
- ☐ I was invited for this, but....

- ☐ Another reason, namely:

How did the process start?

How did you receive the result?

Invitation — Result

Assignment 1C: How I experienced the preventive examination/screening

My reason for participating

Please indicate on the timeline above what the preventive examination/ screening process looked like.

What were the steps in the process?
How long did it take?
What was important for you?

Example

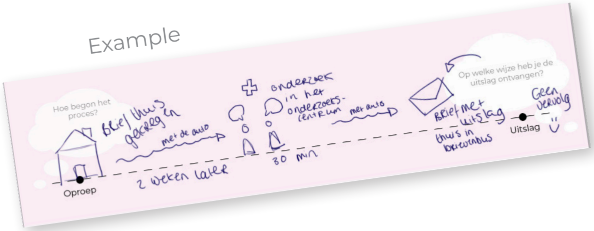

ASSIGNMENT 2: MY VIEW ON THE BREAST CANCER POPULATION SCREENING

The breast cancer population screening is intended to detect at an early stage if a woman has breast cancer. This increases the chance of successful treatment. Also, in many cases, less invasive treatment is required. Currently, women aged 50 to 75 are invited for the screening every two years.

The image below shows the process of the population screening:

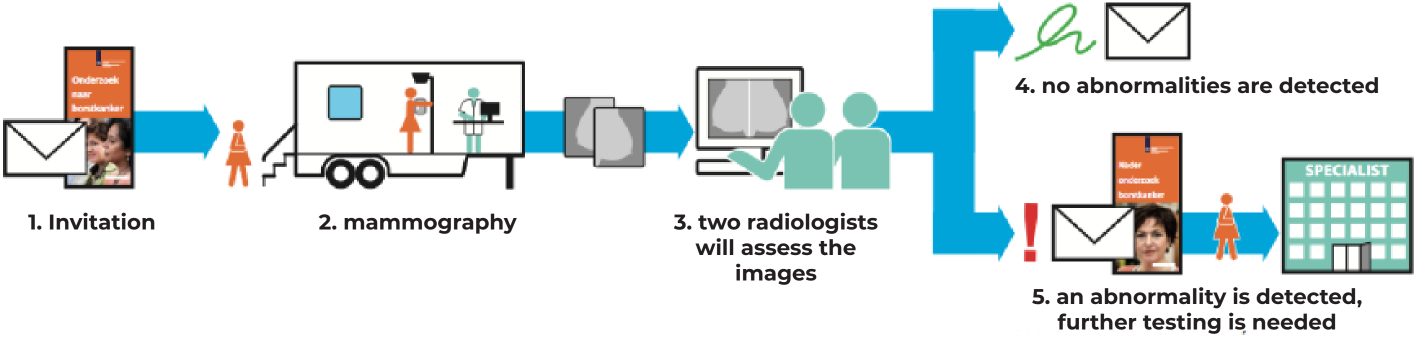

Bron: RIVM

Assignment 2A: The breast cancer population screening evokes these feelings in me:

Friendly, calm, unpleasant, amazed, nervous, warm, encouraging, helpless, worried, predictable, pleased, stunned, happy, uncertain, sad, optimistic, open, stimulated, fascinated, curious, uncomfortable, secure, pleasant, fearful, longing, painful, disinterested, relieved, unhappy, certain, familiar, nervous, hopeful, neutral, honest

Assignment 2B: If I am invited, I will/will not participate, because....

Amsterdam UMC
