# Supplementary material for: Communicating the results of risk-based breast cancer screening through visualizations of risk: a participatory design approach
Source: BMC Med Inform Decis Mak. 2024 Mar 18;24:78. doi: 10.1186/s12911-024-02483-6 (PMC10949766; doi:10.1186/s12911-024-02483-6)
Supplement: Supplementary file 3 — Supplementary Material 3. [file 12911_2024_2483_MOESM3_ESM.pdf]

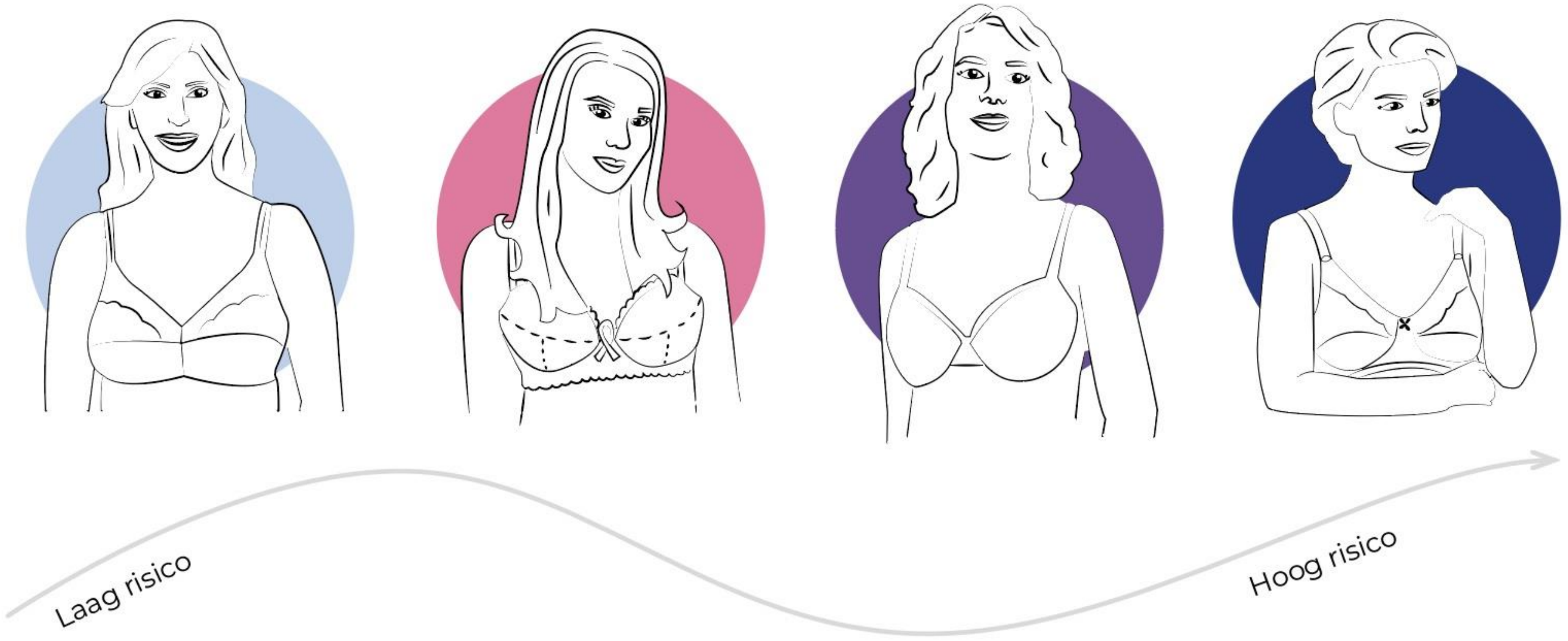

Visualization a

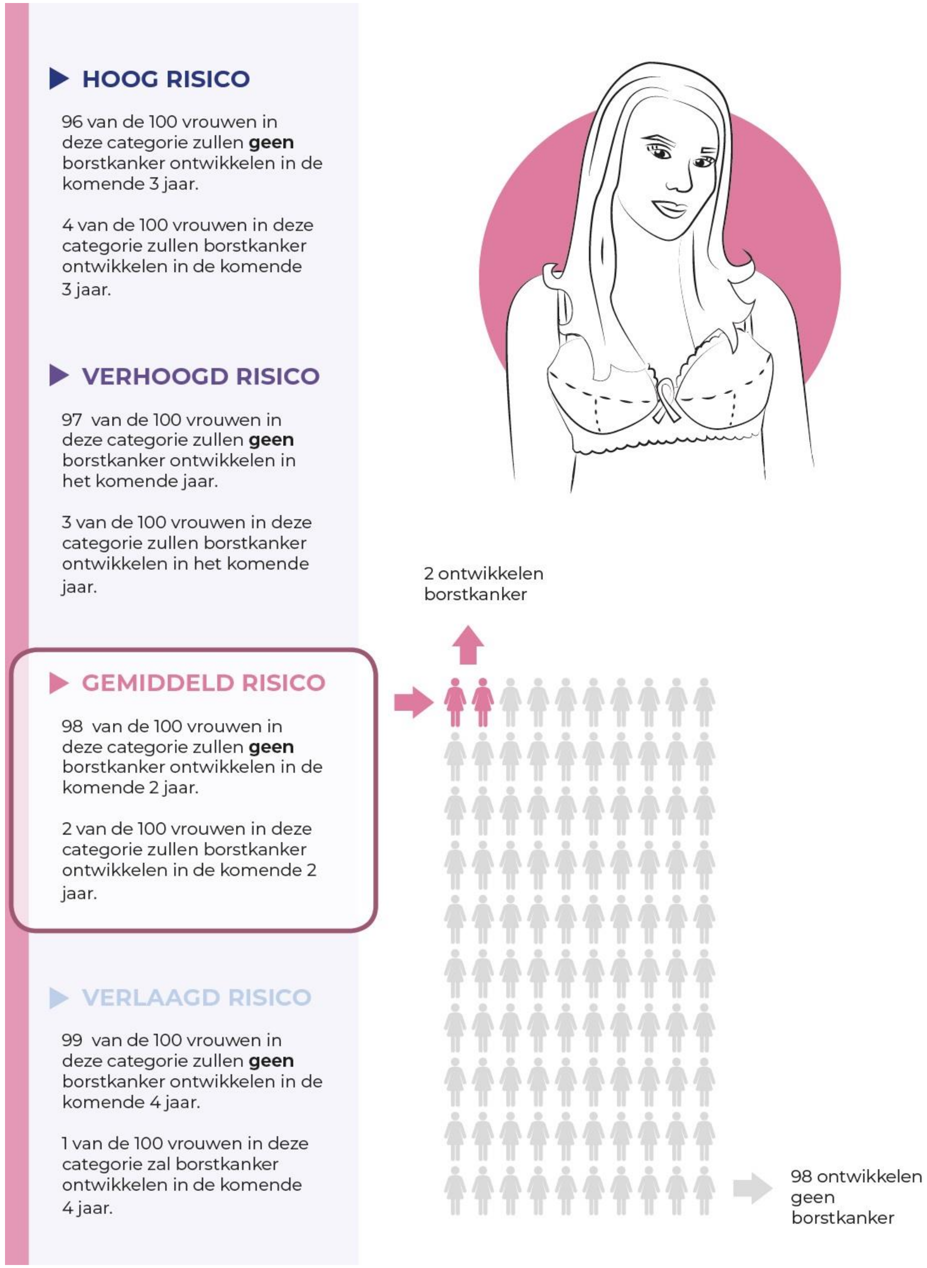

Visualization b

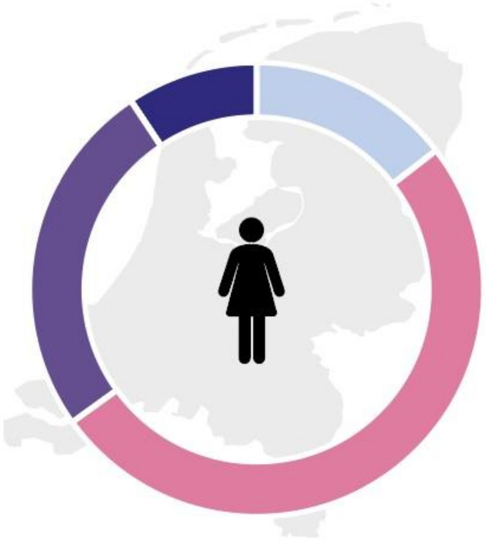

Visualization c

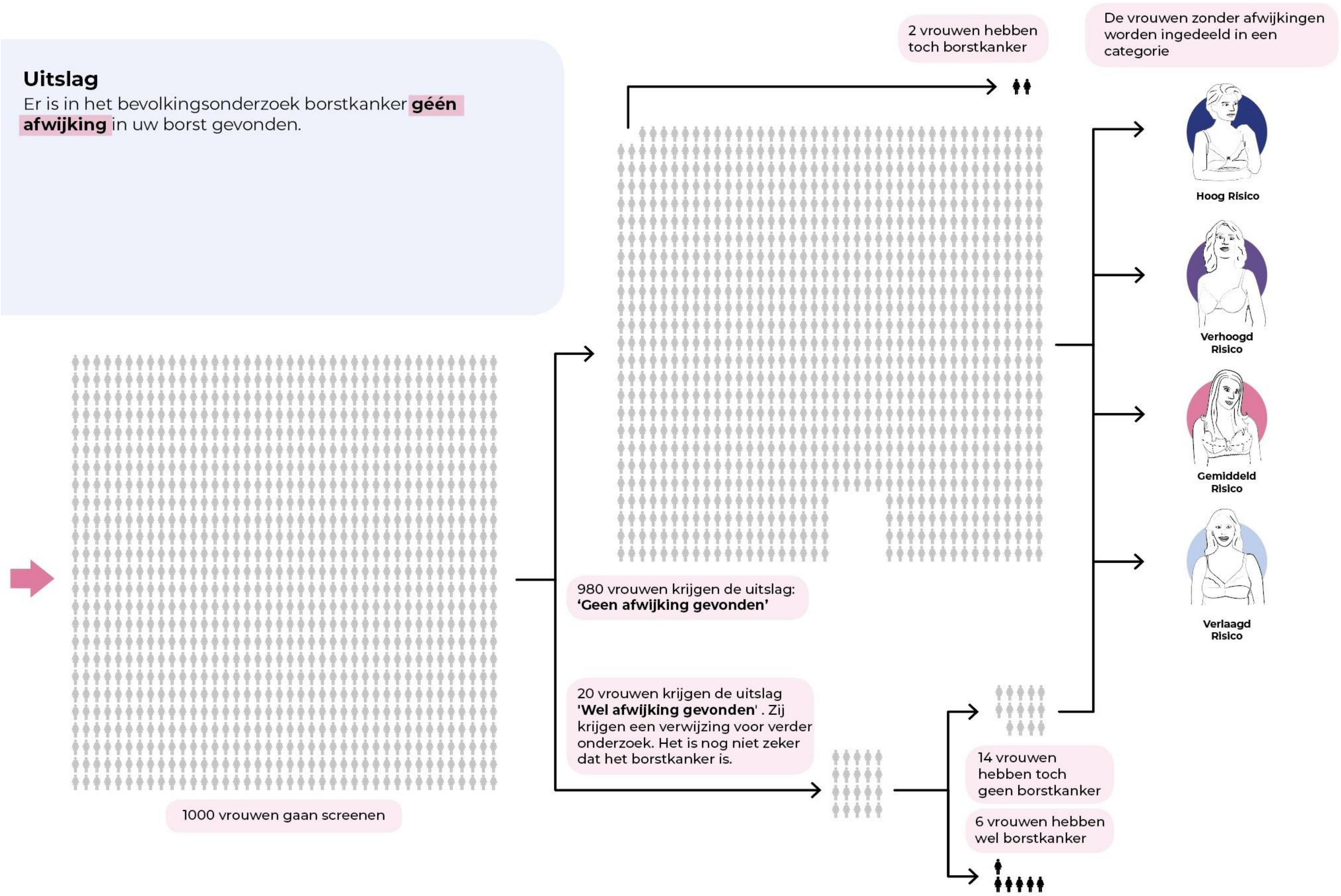

Visualization d

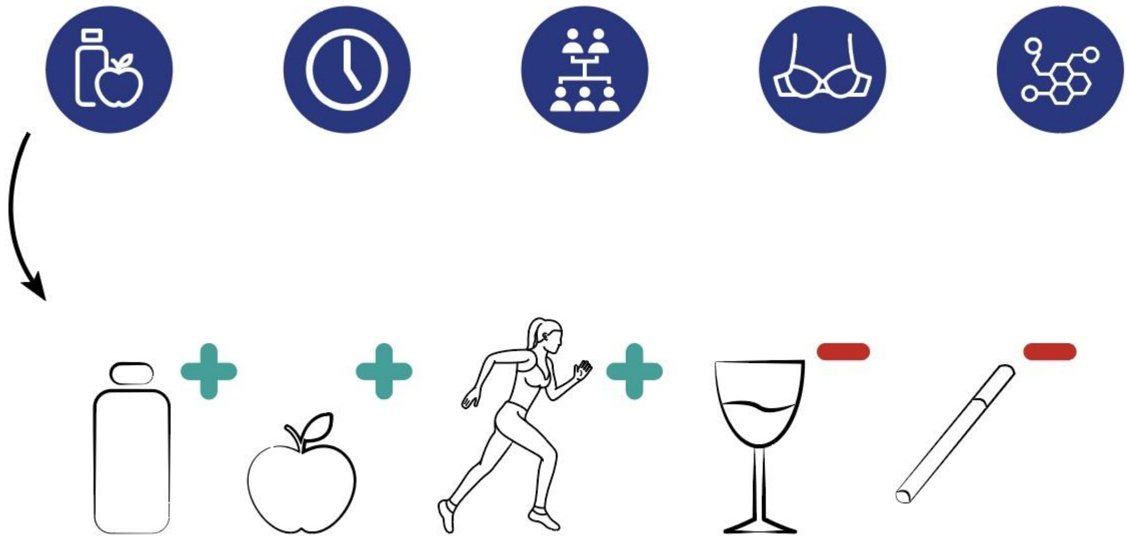

Visualization e

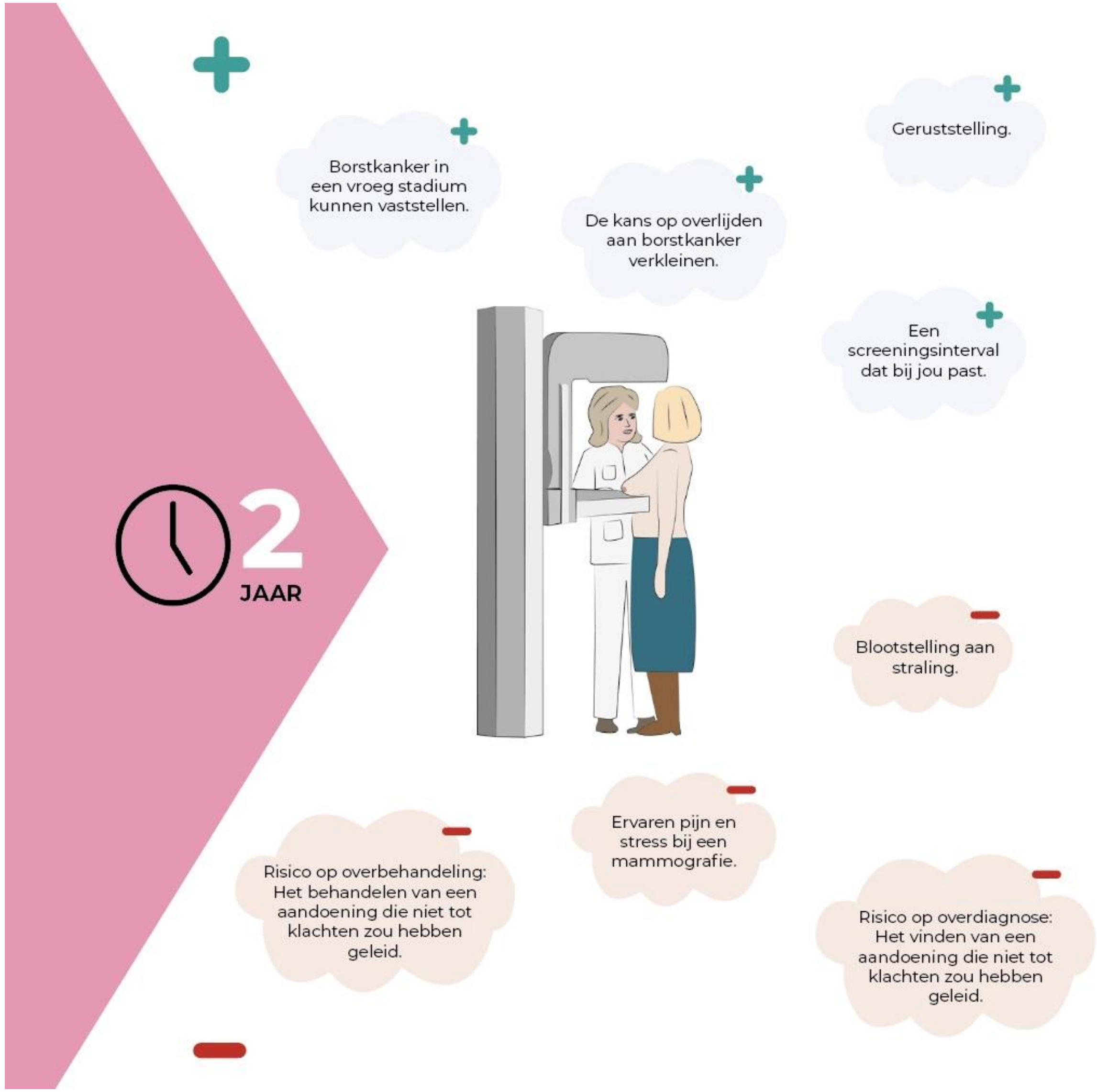

Visualization f
