# Supplementary material for: Communicating the results of risk-based breast cancer screening through visualizations of risk: a participatory design approach
Source: BMC Med Inform Decis Mak. 2024 Mar 18;24:78. doi: 10.1186/s12911-024-02483-6 (PMC10949766; doi:10.1186/s12911-024-02483-6)
Supplement: Supplementary file 4 — Supplementary Material 4. [file 12911_2024_2483_MOESM4_ESM.pdf]

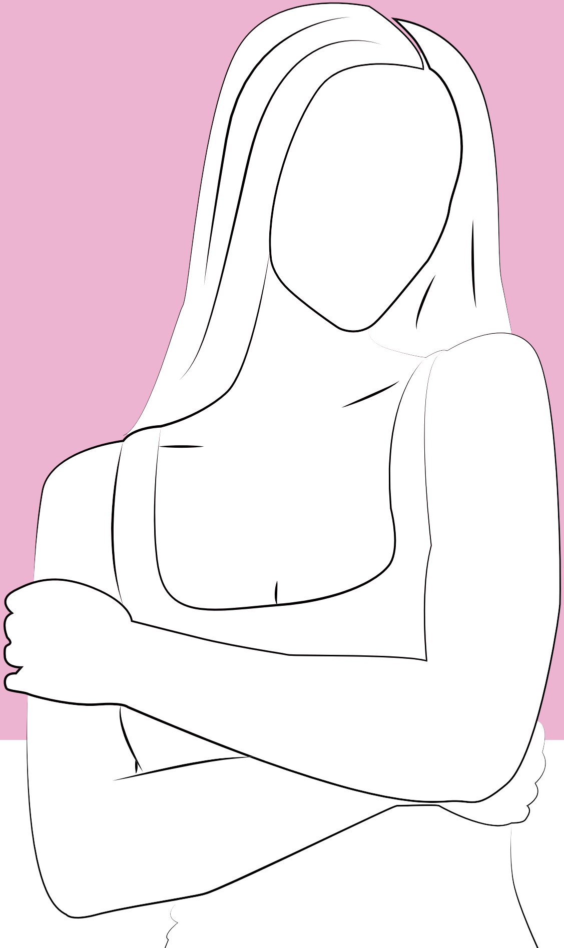

BREAST CANCER POPULATION SCREENING

My experiences and expectations

Amsterdam UMC

INTRODUCTION

Thank you for participating in the session on breast cancer population screening, which will be risk-based in the future. This booklet consists of two short digital assignments for you to complete in preparation for the session. There are no right or wrong answers. We are curious about your own experiences and opinion! Complete the assignments by clicking on the light blue text boxes and typing your answer there. This is only possible on a laptop or computer, not on a phone. Do not forget to save the PDF once you have completed all parts of the assignments.
If you have any questions, you can always contact us:

06-12345678
example@amsterdamumc.nl

06-12345678
example@amsterdamumc.nl

About me

Name:

Age:

Place of residence:

Education/profession/background:

Participating in the breast cancer population screening means to me:

ASSIGNMENT 1: MY EXPERIENCE WITH THE BREAST CANCER POPULATION SCREENING

The breast cancer population screening is intended to detect at an early stage if a woman has breast cancer. This increases the chance of successful treatment. Also, in many cases, less invasive treatment is required.

Assignment 1A:
My reason to participate in breast cancer screening:

Assignment 1B: The breast cancer population screening evoked these feelings in me:

Circle the words that apply

Friendly
calm
unpleasant
amazed
nervous
warm
encouraging
helpless
worried
predictable
pleased
stunned
happy
uncertain
sad
optimistic
open
stimulated

fascinated
curious
uncomfortable
secure
pleasant
fearful
longing
painful
disinterested
relieved
unhappy
certain
familiar
nervous
hopeful
neutral
honest

RISK-BASED SCREENING

Currently, women aged 50 to 75 are invited every two years to participate in the breast cancer population screening. In the future, it will be possible to have a risk-based screening. In so-called 'risk-based screening' women are categorized into risk categories. Each risk category has a corresponding screening interval and screening method. Women at low risk will be invited less often and women at high risk will be invited more often. Also, women at high risk may receive an MRI instead of a mammogram. A woman's risk category depends on various factors and is determined by means of a mammogram and a survey.
The image below shows what the process of risk-based screening will look like:

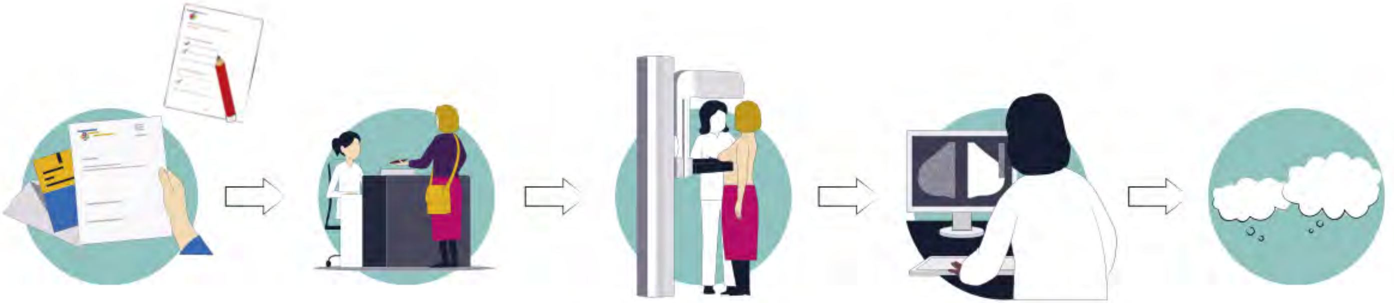

1. Invitation + survey

2. Arrival at screening center

3. Mammography

4. Analyses

5. The result
categorization into a risk category (low-, medium-, high risk) when no abnormality is detected.

ASSIGNMENT 2: SUPPOSE YOU ARE PARTICIPATING IN RISK-BASED SCREENING

Assignment 2a:
Think of 3 advantages and 3 disadvantages of risk-based screening, as explained on the previous page

Advantage 1:

Disadvantage 1:

Advantage 2:

Disadvantage 2:

Advantage 3:

Disadvantage 3:

Assignment 2b:
Information like the risk category and the screening interval will be communicated in the result letter. Describe in the text box below what information you need. What is important for you to know?

Amsterdam UMC
