# Supplementary material for: Communicating the results of risk-based breast cancer screening through visualizations of risk: a participatory design approach
Source: BMC Med Inform Decis Mak. 2024 Mar 18;24:78. doi: 10.1186/s12911-024-02483-6 (PMC10949766; doi:10.1186/s12911-024-02483-6)
Supplement: Supplementary file 5 — Supplementary Material 5. [file 12911_2024_2483_MOESM5_ESM.pdf]

Interview codes

Category	Final code in axial coding	Example
Trade-off regarding participation	Trade-off regarding breast cancer screening	It's good, you know. That's.. that's as it should be.
	Trade-off regarding risk-based breast cancer screening	I just assume that there are people who studied for this.
Risk perception	Risk factors	That you can have such a gene. That it is very common in some families.
	General breast cancer risk	I thought 1 in 3
	Individual breast cancer risk	I hope very small
Knowledge and beliefs	Knowledge and beliefs regarding breast cancer	I'm thinking about chemo, breast amputation, and being ill. And also sadness.
	Knowledge and beliefs regarding breast cancer screening	I know there is such a bus, but I also wonder whether that is still the case.
	Knowledge and beliefs regarding risk-based breast cancer screening	In my opinion, the risk factors would then be identified even sooner
Information needs	Information needs regarding risk-based breast cancer screening	Well, what is the reason for that indication? So why are you in low?
	Information needs regarding receiving the results	If I am in a high category, I would like to have a personal conversation.
Attitude	Attitudes regarding breast cancer screening	I think that's just the unpleasantness.
	Attitudes regarding risk-based breast cancer screening	Well, I think that's very positive.
Intention	Intention regarding breast cancer screening	I would participate in the population screening anyway.
	Intention regarding risk-based breast cancer screening	Yes, I see it as an opportunity of course.
Values and emotions	Values and emotions regarding breast cancer	You hear about cancer, I don't like the word, but okay.
	Values and emotions regarding breast cancer screening	Not so much like, oh how scary or anything like that.
	Values and emotions regarding risk-based breast cancer screening	It might cause anxiety for people who have to return quickly.

Note. The subcodes are not shown in this Table.
