# Supplementary material for: Communicating the results of risk-based breast cancer screening through visualizations of risk: a participatory design approach
Source: BMC Med Inform Decis Mak. 2024 Mar 18;24:78. doi: 10.1186/s12911-024-02483-6 (PMC10949766; doi:10.1186/s12911-024-02483-6)
Supplement: Supplementary file 6 — Supplementary Material 6. [file 12911_2024_2483_MOESM6_ESM.pdf]

BREAST CANCER SCREENING

RESULT

No abnormalities found | Risk category 3

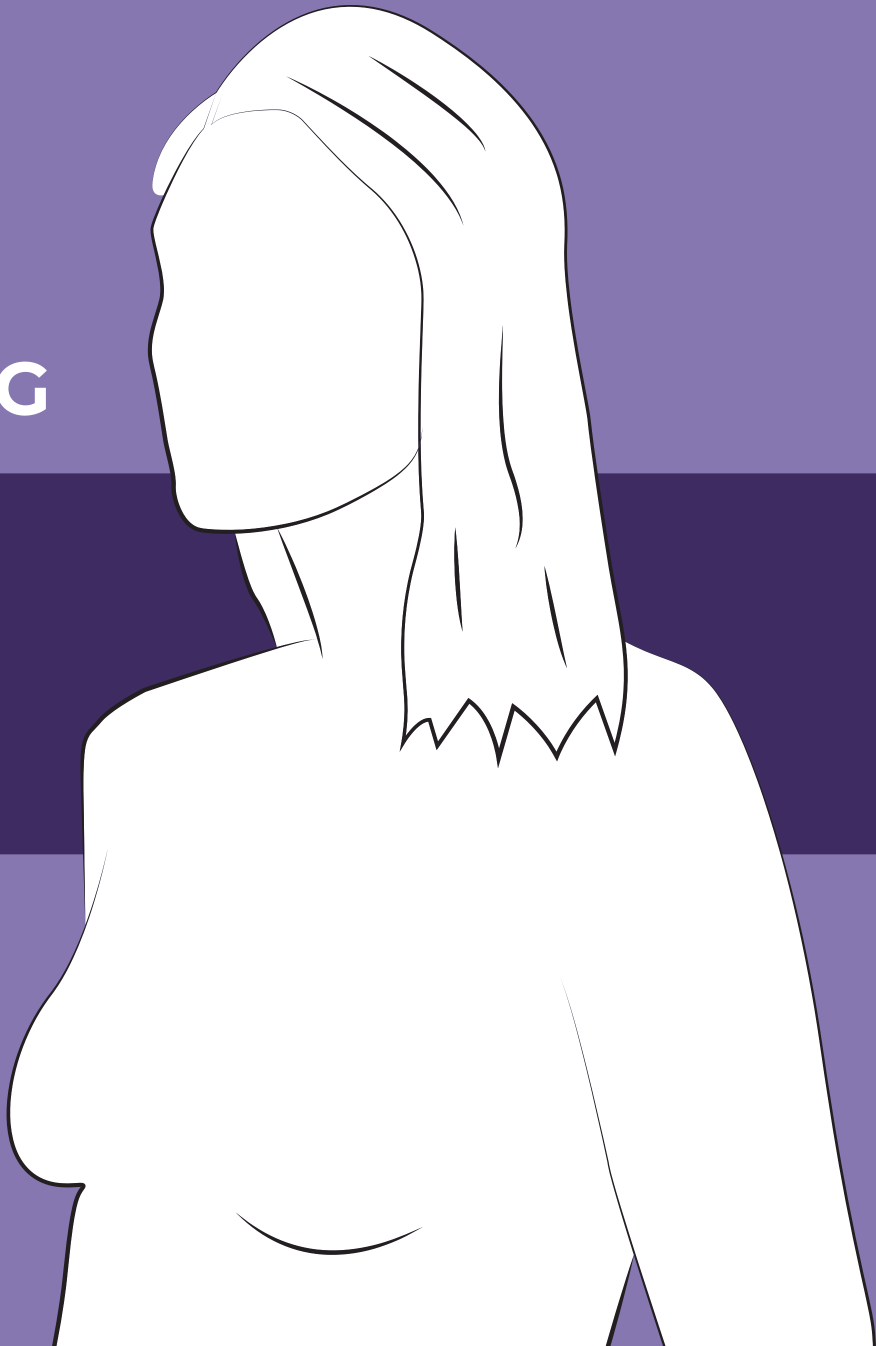

RESULTS OF RISK-BASED BREAST CANCER SCREENING

What are the screening results?

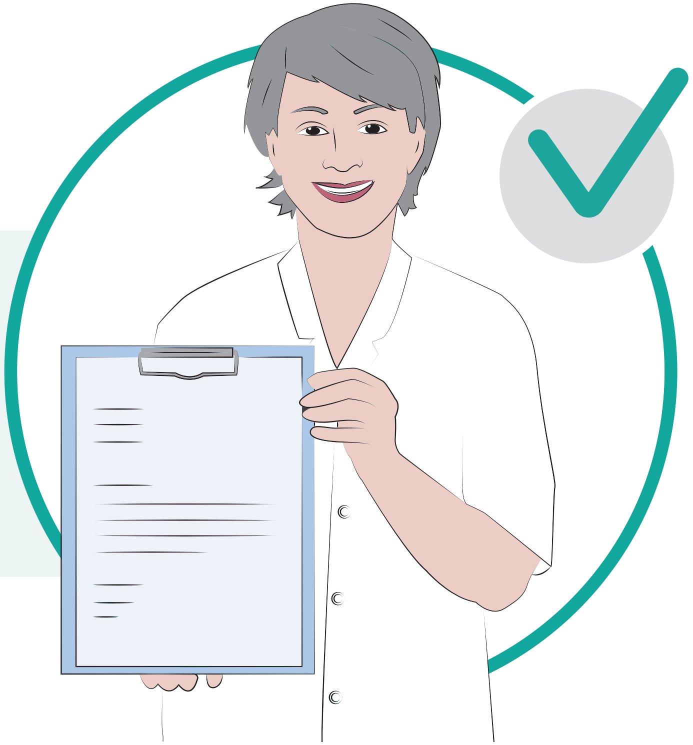

Results

No abnormalities found
No indications of breast cancer were found.

This means that no further examination is required in the hospital.
You will receive another breast cancer screening invitation after 1 year.

Reliability

This result is almost certain. There is always a small chance that an abnormality will be missed.

When 1000 women are screened, 990 will receive the result that no abnormality was found.

- Of this group of 990 women, 989 received the correct result: they do not have breast cancer.
- Of this group of 990 women, 1 woman receives the wrong result: an abnormality has been missed.

MY RISK CATEGORY

No abnormality, what does this mean for me?

A risk category
It is important that we invite all women at the right moment for the next breast cancer screening. That is why we categorize you into a risk category. This happens to all women in whom no abnormality was found.

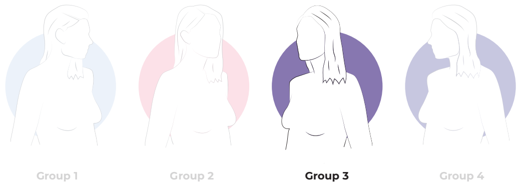

Group 3
Based on the questionnaire and the screening result, you have been categorized in risk category 3. This means you have a medium-high risk of developing breast cancer.

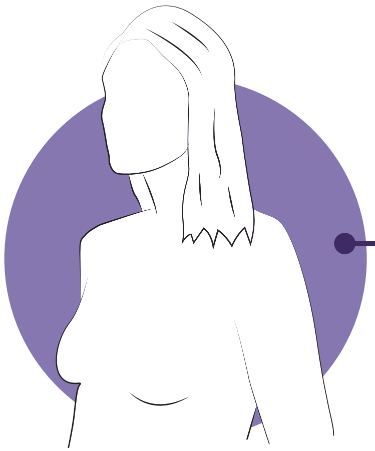

Group 3
Medium-high risk

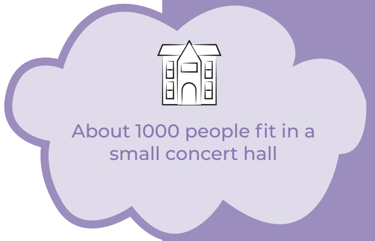

Medium-high risk

Risk of breast cancer within now and 5 years

- Within the next 5 years about 980 out of 1000 women in this category will not develop breast cancer
- Within the next 5 years about 20 out of 1000 women in this category will develop breast cancer

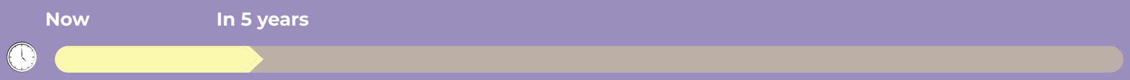

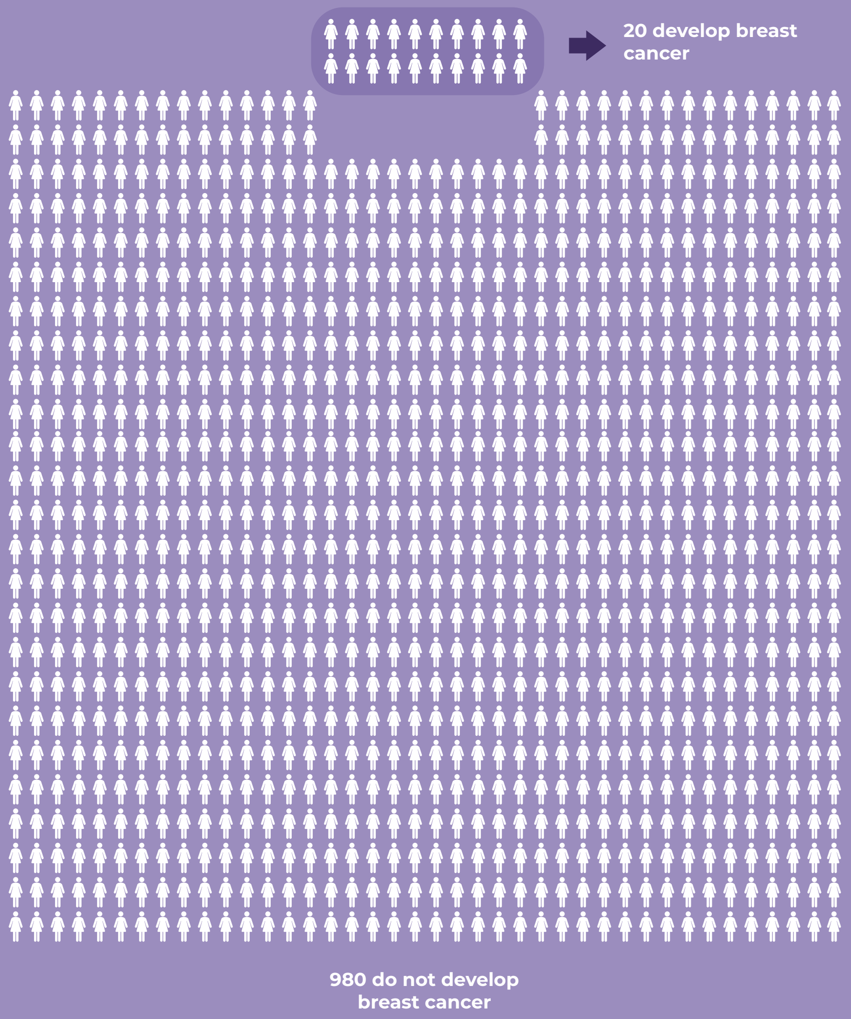

SCREENING PLAN

When do I need to come back and what is the procedure?

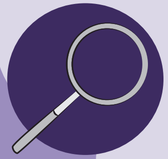

BREAST SELF-EXAMINATION

Breast self-examination

In the period between the screening rounds, you can examine your breasts yourself. You will then know what is normal for you and you will be able to feel or see a change in your breasts more quickly.

Please contact you General Practitioner if you notice an abnormality.

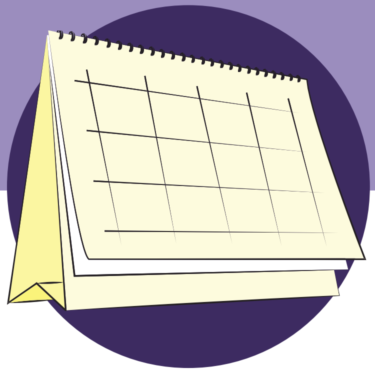

1 YEAR

Based on your risk category, you will receive another breast cancer screening invitation after 1 year. You will then have a mammogram. Each time you participate, we again evaluate in which risk category you are categorized. If your risk category changes, the time between screenings will also change.

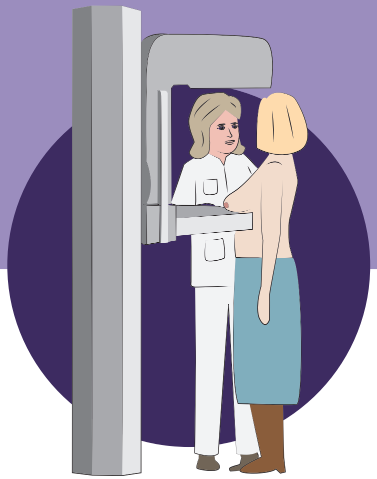

A MAMMOGRAM

A mammogram is an X-ray of the breast that is taken to detect abnormalities. A lab technician takes these pictures. Your breasts are placed on a plate and compressed by another plate. When your risk category changes, the screening method may also change.

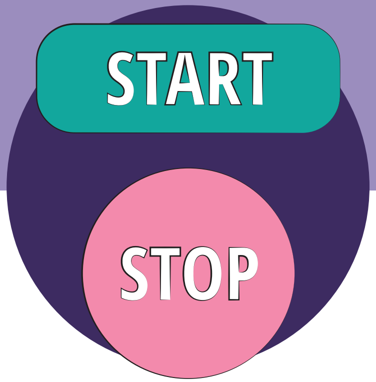

SCREENING FROM 45 TO 75 YEARS

Based on your risk category, you start screening at age 45. You stop screening at the age of 75. When your risk category changes, these ages may also change.

RISK FACTORS

Which risk factors influence the risk of developing breast cancer?

Sources
Dutch Breast Cancer Association
International Agency for Research on Cancer – WHO
Pink Ribbon

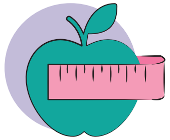

Lifestyle

Lifestyle is a factor that you can influence yourself. The healthier your lifestyle, the smaller the chance of developing breast cancer. Accordingly, this is our advice:

- **Maintain a healthy weight:** a healthy weight reduces the risk of breast cancer. The Body Mass Index (BMI) indicates whether you have a healthy weight.
  - **Eat healthy:** Lots of vegetables, fruit, whole grains, beans, oil, and nuts. Avoid too many dairy products, sweets, snacks, and soft drinks.
  - **Drink plenty of water:** About 2 liters a day. This amounts to about 8-10 glasses of water.
  - **Exercise regularly:** Exercise can be a specific sport, but your daily activities also count as exercise. Find a way of exercising that suits you.
- **Do not smoke:** Smoking increases the risk of breast cancer and also causes other cancers.
- **Drink in moderation:** Preferably, do not drink alcohol at all.

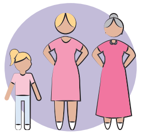

Age

Older women have a higher risk of breast cancer.

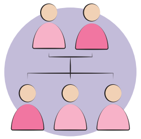

Breast cancer in the family

Women with a lot of family members who had breast cancer have a higher risk of developing breast cancer.

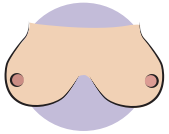

Breast density

Women with dense breast tissue (a lot of glandular and connective tissue and little fatty tissue) get breast cancer more often than average. Breast density can be determined by a mammogram or MRI.

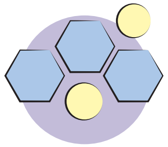

Hormone balance

- Women who have never been pregnant have a higher breast cancer risk.
- Women who became mothers later in life have a higher breast cancer risk.
- Not breastfeeding or breastfeeding for a short period (4 months) can lead to an increased breast cancer risk.
- An early menstrual period (12 years or younger) increases breast cancer risk.
- Women with late-onset menopause (55 or older) have a higher breast cancer risk.

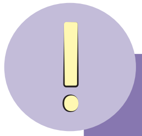

These are the most important risk factors for you:

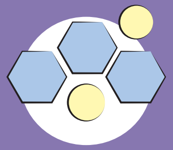

Hormone balance

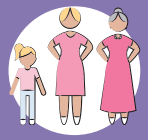

Age

GENERAL INFORMATION

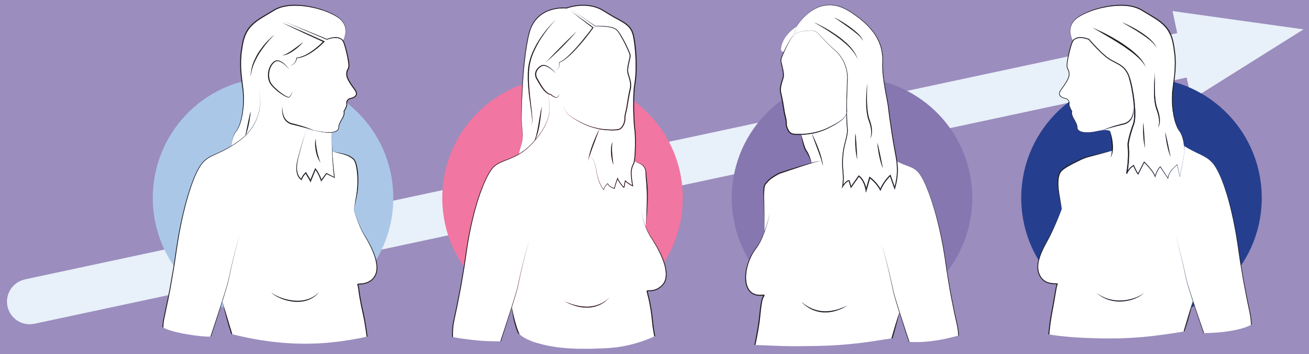

- What does risk-based screening entail?
- What are the benefits and harms of
- risk-based screening?

INTRODUCTION

What is risk-based screening?

Risk-based screening
Early detection of breast cancer by means of breast cancer screening ensures that fewer people die from breast cancer. Population screening is constantly being improved. Because screening can also have harmful consequences, and resources are limited, you cannot simply increase the amount of screening. It is important to use resources for the group that will benefit the most from it. This is how risk-based screening emerged.

What is risk-based screening?
Why do some women develop breast cancer while others do not? Multiple risk factors are involved in this. Risk-based screening enables us to measure these risk factors. This allows us to know who is more at risk and who is less at risk. Women are then categorized into different risk categories with a higher or lower breast cancer risk. These categories differ in terms of the time between two screening rounds, screening method and starting and stopping age.

Why risk-based screening?
Just like taking medication, screening also has several harmful consequences. It is important that these harmful consequences are in balance with the breast cancer risk. After all, you don't take medication if you don't need it. Risk-based screening provides this balance.

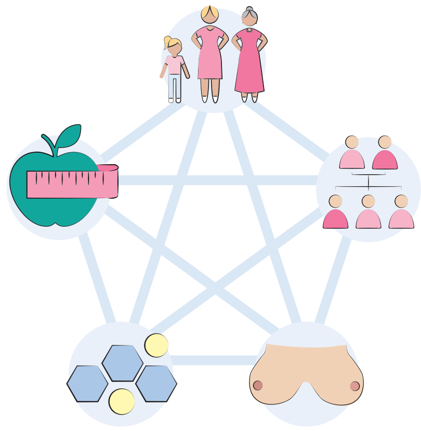

Breast cancer risk is caused by multiple risk factors

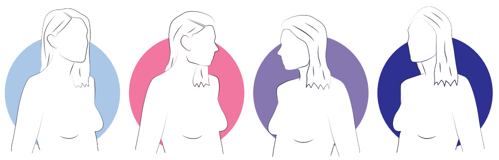

The risk factors determine the risk category and a corresponding screening plan

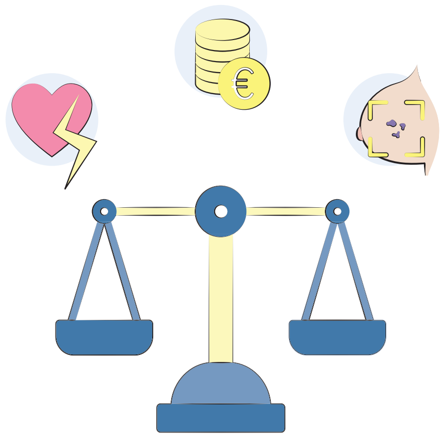

Following this screening plan results in a good balance between the benefits and harms of screening, the available resources, and the ability to properly detect abnormalities.

RISK-BASED CANCER SCREENING

How does it work?

From invitation to result
The figure below shows the procedure of the risk-based breast cancer screening.

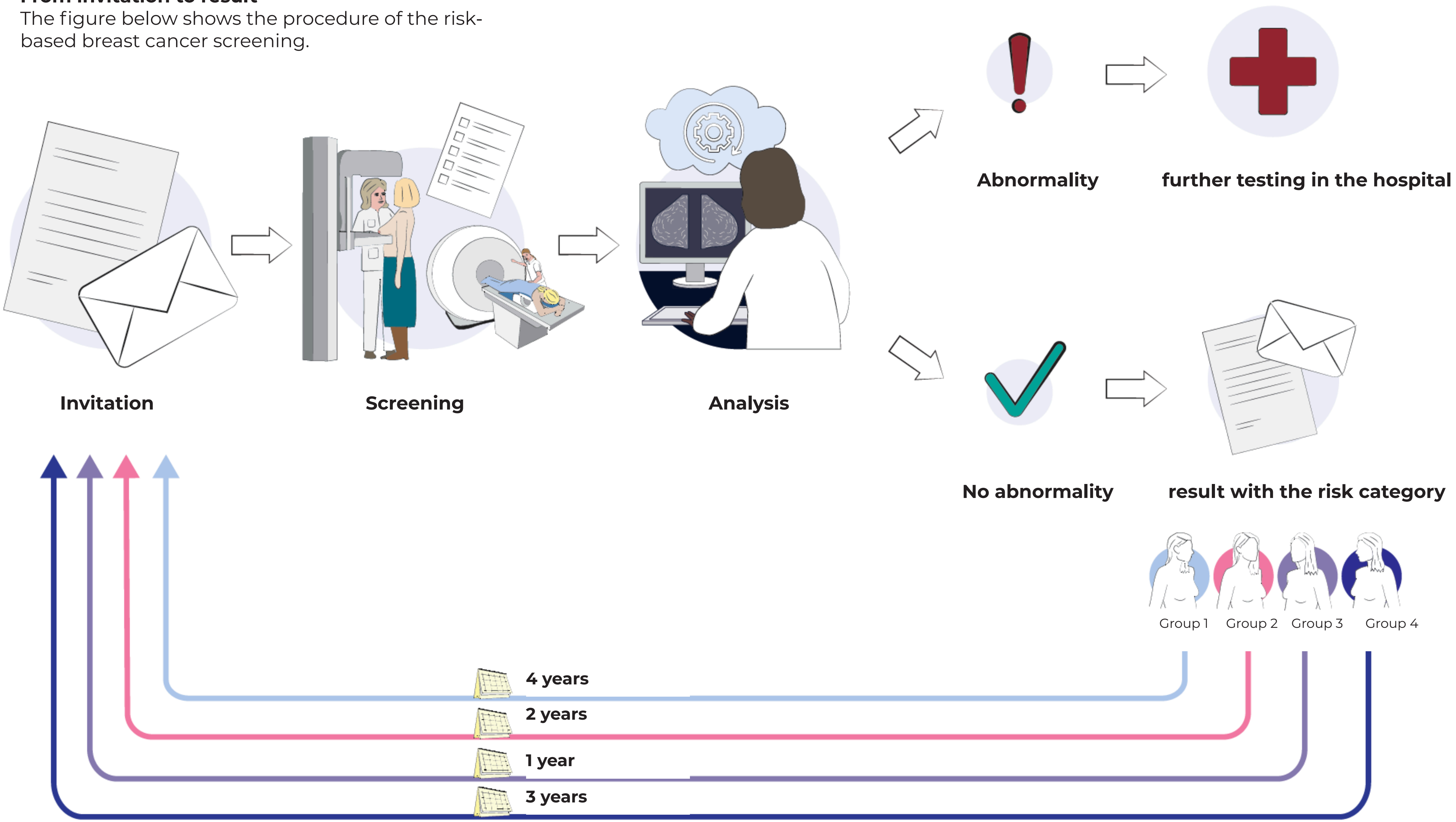

BENEFITS AND HARMS

What are the benefits and harms of risk-based cancer screening?

What are the benefits?

- Breast cancer can be detected at an **early stage.**
- **Less intensive treatment** when breast cancer is detected at an early stage.
- **Reassurance** when no abnormalities are found.
- **Lower risk** of dying from breast cancer.
- Following a **screening plan** appropriate to the breast cancer risk.

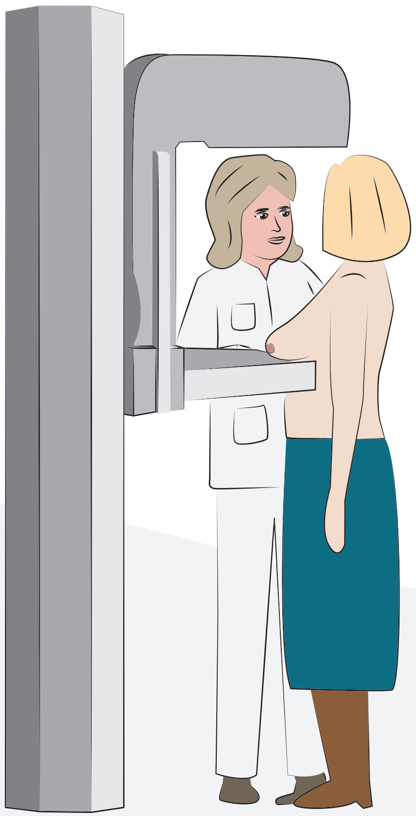

What are the harms?

- **Pain and stress** during a mammography.
- Exposure to **radiation.**
- Risk of receiving treatment for a condition that **would not have led to any physical complaints.**
- Risk of **false reassurance:** the results do not provide 100% certainty that nothing is wrong.
- Risk of **unnecessary fear:** someone can be referred for further testing, even though nothing is wrong.
- Risk of a **faulty risk categorization**.

FOR FURTHER QUESTIONS:

info@riskbasedcancerscreening.com

06-12345678

www.riskbasedcancerscreening.com
